# First evaluation of the emotional picture set of self-injury images (EPSI) using psychophysiological and self-report measures

**DOI:** 10.1186/s40479-025-00304-4

**Published:** 2025-07-12

**Authors:** Sarah-Louise Unterschemmann, Erik Malte Mueller, Silke Lux, Alexandra Philipsen, Marcel Schulze

**Affiliations:** 1 Department of Psychology, University of Marburg, Gutenbergstr.18, Marburg, 35032 Germany; 2 Department of Psychiatry and Psychotherapy, University of Bonn, Venusberg-Campus 1, Bonn, 53127 Germany

**Keywords:** NSSI, Picture Set Evaluation, Emotional Regulation, Borderline Personality Disorder, Psychophysiology

## Abstract

**Background:**

Nonsuicidal self-injury (NSSI) has been identified as one of the most important predictors of suicidal ideation and attempts. Nevertheless, previous research on NSSI has been limited, with different experimental designs leading to inconsistent findings regarding its underlying factors. This is especially true for time-related processes of NSSI. To address this, a standardized set of symptom-specific emotional stimuli was developed (Emotional Picture Set of Self-Injury Images, EPSI), depicting differing stages of NSSI. This study offers a first evaluation of the EPSI picture set as a measurement of emotional reactivity by using emotional self-reports and psychophysiological measures in a sample of healthy participants.

**Method:**

The EPSI picture set, which includes images with neutral objects, self-injury objects, and self-injury scenes, was presented to *N* = 64 (age: 22.42 (1.4) years, sex: 18 m/ 46f) healthy participants. Emotional reactivity was assessed using emotional self-reports (Self-Assessment Manikin, SAM-Rating) for valence and arousal dimensions. Additionally, psychophysiological measures including skin conductance response, heart period, and the acoustic evoked startle response were recorded.

**Results:**

Overall, the intraclass correlation coefficient and item-total correlation were sufficiently high, indicating good reliability at both the picture and category level. Participants rated self-injury pictures as significantly more unpleasant and arousing compared to pictures showing objects of self-injury and neutral objects. The skin conductance response to pictures with scenes of self-injury was significantly increased compared to pictures with neutral objects, as was heart period deceleration.

**Conclusions:**

This initial evidence suggests that EPSI elicits distinct emotional responses and may serve as a valid measure for studying the process of NSSI. Future research could investigate the EPSI as a standardized measure, particularly in patients with NSSI and borderline personality disorder, to validate its clinical utility and gain insight into its psychophysiological mechanisms.

**Supplementary Information:**

The online version contains supplementary material available at 10.1186/s40479-025-00304-4.

## Background

The lifetime prevalence of non-suicidal self-injury (NSSI) worldwide is estimated to be around 18% [68, 81, 99]. NSSI has been identified as a robust risk factor for suicidal ideation and attempts [87]. In addition to direct medical treatment costs of NSSI-related injuries [61, 101], it leads to long-term functional impairment [41]. NSSI appears as a trans-diagnostic symptom in a variety of mental disorders (e.g., personality disorders, mood disorders [3, 112]), and has been listed in the DSM-V as a condition in need of further study [43, 44, 113]. It encompasses any behavior intended to harm oneself and to deliberately destruct body tissue, as opposed to indirect self-harm such as drug abuse [71, 72]. Specifically, it describes a behavioral cluster including biting, cutting, burning the skin, hair, nails, self-punching, self-scratching, or head-banging [20].

Even though therapy seems to be partially successful [22, 75, 76, 102], complete remission remains rare with some studies reporting a total absence after therapy in only 25% [85]. These rates are alarmingly low, especially considering that there is no specific pharmacological intervention for NSSI [6, 67, 74]. Biological factors contributing to NSSI are still unknown, with some evidence pointing towards a dysregulation of psychobiological systems of emotional processing [38, 47, 108].

Still, inconsistent findings have been reported regarding altered emotional processing in NSSI: While some studies observed hypoarousal, others found hyperarousal when examining physiological measures and subjective reports [40, 73]. Additionally, a considerable number of recent studies have reported no significant physiological differences at all [23, 46, 65, 88, 89].

While some of these contradictory results may be explained by the substantial heterogeneity in stimuli and paradigms used, there is currently no standardized stimulus set available for studying the processing of disorder-specific stimuli in NSSI. Most studies examining emotional processing in NSSI have employed different paradigms, including (1) distressing tasks such as psychosocial stress-tests [23, 46, 73, 82, 107], (2) imagination tasks involving acts of self-injury or emotional scripts [17, 40, 53], and (3) the induction of painful or aversive stimuli, mirroring acute self-injury [80, 86, 91, 92]. Studies involving NSSI-specific content used stimuli that were neither standardized nor publicly accessible [29, 30, 39, 43, 44].

While some efforts have been made to develop standardized stimulus sets—such as a subset of the prominent International Affective Picture System (IAPS; NIMH Center for the Study of Emotions and Attention, 1995IAPS; [14, 15]) rated specifically for NSSI and borderline personality disorder—these have shown limited success in eliciting specific emotional reactions in clinical populations. For example, in empirical validation, the subset of the IAPS developed by Sloan et al. [95] for NSSI and borderline personality disorder failed to evoke distinct emotional responses in individuals with borderline personality disorder compared to healthy controls [27]. While standardized picture sets like IAPS are widely utilized in research and have demonstrated strong validity and reliability for general emotion studies [12, 16] and certain psychopathologies [42, 62, 70, 96], they currently appear to lack relevance to the specific themes and triggers associated with NSSI.

Moreover, NSSI itself is a complex behavior, with many factors contributing to the disorder, including immediate temporal aspects, which complicate its study when using different emotional stimuli that capture only a moment of emotional reactivity rather than elements of the entire process [45]. Kaess et al.’s [47] state-trait model of NSSI describes states and traits within a temporal framework of the genesis of NSSI. Traits are stable, enduring characteristics of behavioral and biological functioning, which may not be directly linked to NSSI but rather reflect functional abnormalities related to the behavior. For example, these include biological representations of adverse childhood experiences, alterations in fronto-limbic neural systems that regulate emotional processing, cognitive control, and reward systems,as well as changes in the autonomic nervous system and pain system [25, 50, 51, 64, 80, 94, 103]. Consequently, these traits may indirectly influence behavior, for instance through a general increase or decrease in emotional reactivity to emotional stimuli, as reflected by physiological hyperarousal or hypoarousal.

A state, on the other hand, reflects a current situation of clinical manifestations [47]. For NSSI, this includes the states directly before, during, and after NSSI events, which, due to ethical constraints, have only been tentatively researched to date. Studies so far suggest that different states may occur along the timeline of NSSI, with differential effects being highly time-dependent (e.g., the differential effects of pain stimulation in NSSI and healthy participants occurring immediately after the stimulation) [86]. Meanwhile, these immediate effects may also serve as maintaining factors of the disorder [71]: NSSI directly leads to a reduction in stress levels (independent of tissue injury), with seeing blood enhancing the stress-dampening effect and a general decrease in subjective arousal, potentially functioning as negative reinforcement [49, 69, 110].

Overall, these results suggest a disorder-specific cascade model of altered emotional, physiological, and cognitive states during the NSSI event, which, in the long term, maintains the dysfunctional behavior. This highlights the importance of studying the stages of the NSSI process itself, rather than only its individual elements (i.e., focusing on the sequence of NSSI rather than solely on the stimulus for self-injury). A dynamic perspective emphasizes that altered emotional processing may be more pronounced or qualitatively different at certain points in the self-injury cycle [71]. Consequently, studying these processes requires stimuli tailored to the NSSI stages—something that current NSSI research has yet to adequately address. Similar approaches have already been employed in the study of other psychopathologies, such as abuse and eating disorder, successfully capturing disorder-specific physiological, cognitive and emotional processes (e.g., craving in drug dependents) [83, 98].

All in all, there is currently no validated, standardized picture stimulus set designed explicitly for studying disorder-specific emotional states in the process of NSSI. In this context, the proposed Emotional Picture Set of Self-Injury Images (EPSI) aims to fill this gap by providing a standardized collection of images depicting various stages of self-injury (SSI), objects associated with self-injury (SIO), and neutral objects (NO) [1]. Neutral objects were chosen based on valence and arousal (low on both dimensions) from an existing database [5]. By including images representing different phases, EPSI enables detailed exploration of the functional and biological mechanisms underlying NSSI at multiple points along its timeline, including the emotional response throughout the process. To immediately study the effects of the NSSI stages, all pictures were presented against a white background (equivalent to the NO pictures), rather than using a more naturalistic approach (as in the IAPS-pictures). To increase the comparability of the SSI pictures, all NSSI acts were depicted in the same position from the perspective of the agent. Objects of self-injury were matched between SIO and SSI pictures.

Thus, the present pilot study aimed to preliminarily validate the emotional responses to the EPSI by assessing both subjective reports and psychophysiological responses in healthy participants. The goals were twofold: (1) to evaluate the reliability and validity of individual pictures through item-level analyses (mean, standard deviation, item-total-correlation) and category comparisons in healthy participants (intraclass-coefficient, Pearson’s correlation); and (2) to explore whether image categories (NO, SIO, SSI) and stages (pre-self-injury, post-self-injury) elicited distinct emotional and physiological reactions. Since the focus of the present study was to provide an initial characterization of the EPSI pictures (which already consisted of 89 images to rate), a direct comparison with the IAPS pictures was not performed.

Using a classical picture viewing paradigm, images were presented randomly while recording skin conductance response (SCR), heart period (HP), and the acoustic startle reflex (ASR). The SCR is presumably mostly influenced by the sympathetic nervous system and often used as an unspecific measure of emotional arousal [13]. Changes in HP, on the other side, have been associated with the parasympathetic system and are interpreted as an indicator of the internal valence and attention system [13, 55]. Meanwhile, the acoustic-evoked startle response (ASR) is an electromyographic reflex influenced by limbic brain structures, and has been also interpreted as a marker of the valence dimension of an elicited emotion, representing its psychological state [37].

The specifics of the paradigm were chosen based on previous studies on the IAPS, and hypotheses were formulated accordingly [13, 24, 54]. Participants rated valence and arousal via the Self-Assessment Manikin (SAM, [12]).

It was assumed that pictures with SSI would lead to higher valence and arousal ratings compared to SIO and NO. To test whether participants successfully discriminated between picture categories, reliability was assessed using item-statistics (mean and standard deviation, item-total-correlation), and validity was evaluated using Pearson’s correlation. Based on prior research with aversive IAPS images (such as those depicting body injury), it was hypothesized that SSI images would produce higher negative valence and arousal ratings, increased SCRs, greater HP deceleration, and stronger ASRs compared to SIO and NO images.

Furthermore, although only healthy participants without a history of NSSI were included, the study examined whether different stages of self-injury would evoke varying responses, assessed habituation effects over repeated presentations of similar stimuli, and explored startle modulation. Statistical testing was performed using multilevel modeling.

## Methods

### Sample

A total of N = 64 students (*n* = 18 men, *n* = 46 women) aged between 18 and 30 years (*M* = 22.42, *SD* = 14) participated in the laboratory study. Participants were recruited through advertisements at the University of Marburg and completed an online survey before beginning the laboratory study. Exclusion criteria were a history of or current self-injury, and a diagnosis of borderline personality disorder. Before starting the study, participants indicated whether they had ever received a psychiatric diagnosis. Participants received credit points for participation and provided informed consent. The study was approved by the local ethics committee of the University of Marburg.

### Questionnaires

Participants completed several questionnaires including the NEO-Five-Factor Inventory (NEO-FFI; Kanning, [48]), the Borderline-Symptom Checklist (BSL-23; [8]), the Positive and Negative Affect Scale (PANAS-SF [19, 106]), and a questionnaire to measure empathy (SPF-IRI, [78]) (see Supplement). The mean score on the BSL-23 was low, with *M* = 1.57 (*SD* = 0.42) on the 4-point Likert scale, indicating low levels of symptoms in the borderline spectrum. A total of 92.2% (59 participants) stated that they never thought of self-injury, while only two participants (3.1%) reported that they had ever strongly considered self-injury.

### Procedure

Before starting the experiment, participants received instructions, electrodes were applied, and a signal check took place. The participants'positions, including the distance to the monitor and room speaker, were standardized. Participants were also instructed to avoid unnecessary movements during the recording time. During the whole experiment, participants were monitored by the experimenter through a camera and could communicate through a microphone. Before and after the experiment, participants were questioned about their well-being and support by a clinician in training was offered if necessary. For additional details regarding the laboratory parameters, please refer to the Supplement.

### The emotional picture set (EPSI)

**Fig. 1:**
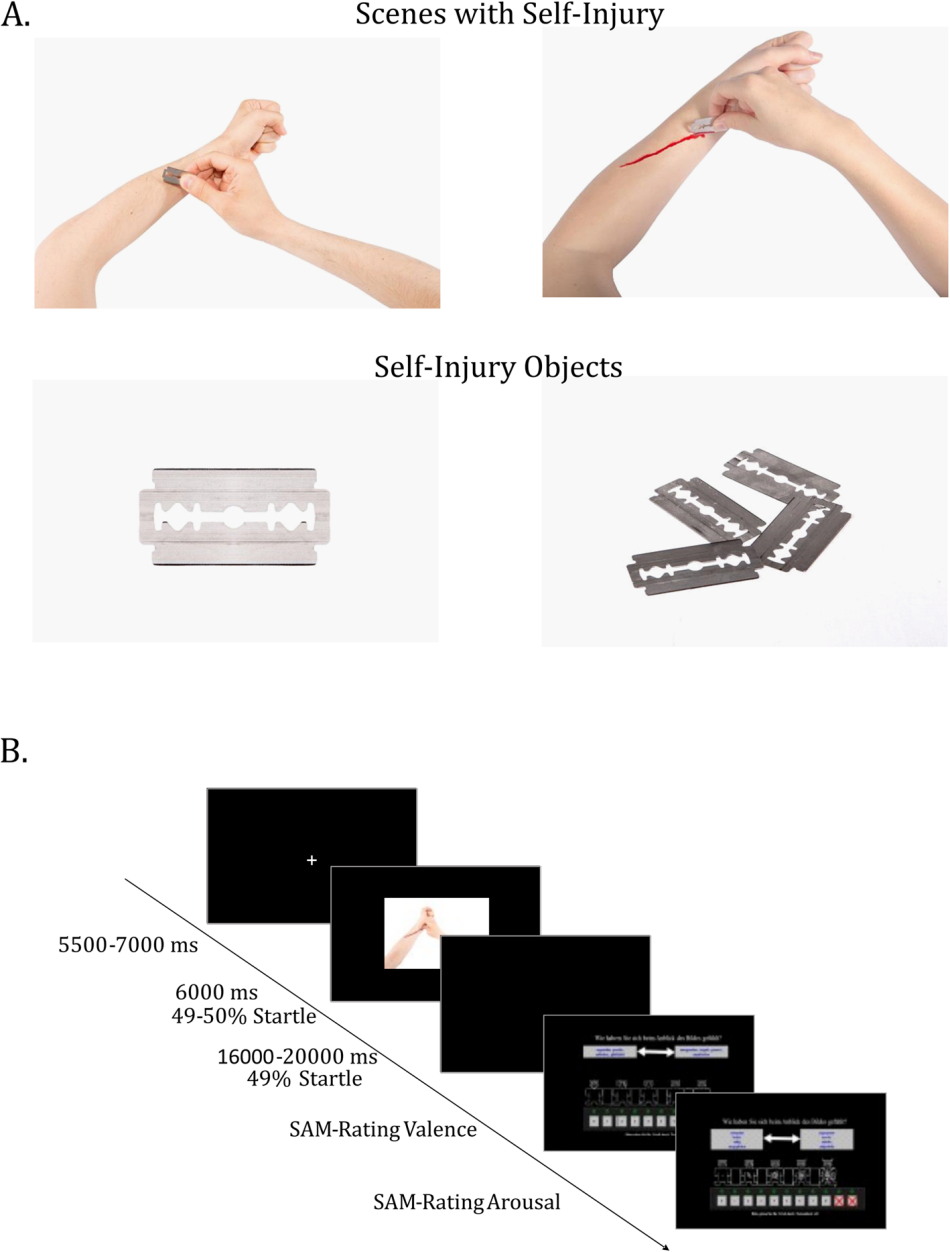
**A** Exemplary pictures of scenes with self-injury (pre-self-injury and post-self-injury) and corresponding self-injury object. **B** Exemplary trial. A total of 89 pictures were presented with 3 picture categories (*n* = 45 images with neutral objects, *n* = 13 images with self-injury objects and *n* = 31 pictures containing self-injury scenes). 49% to 50% of the pictures were presented with an acoustic startle, respectively

The set consisted of 89 pictures presented in randomized order. 45 pictures featuring neutral objects (NO),^1^ 13 pictures depicting self-injury objects (SIO), and 31 pictures showing self-injury (SSI) on the right or left arm were presented (SSI). SSI included explicit scenes of self-injury depicting various methods such as cutting, burning, and incising with different objects on the skin of the inner/outer side of the left/right forearm (e.g., knives, razor blades, wire, iron; Fig. 1A). The SSI varied in the severity of the depicted self-injury, ranging from images with no blood or early stages of self-injury to images with open wounds or advanced stages of self-injury. A total of 13 pre-NSSI pictures and 18 post-NSSI pictures were presented. Male and female actors were used to enhance real-life similarity and identification for male and female participants, increasing the emotional reactivity. Pictures of SIO were matched to the SSI so that the self-injury objects were identical.

### Paradigm

Participants engaged in a classical picture-viewing paradigm, following the methodology outlined by Bradley et al. [10]. The experiment began with a 5-min baseline measurement, after which participants were trained on providing valence and arousal ratings using the Self-Assessment Manikin [12]. The white noise burst (95 dB SPL, 500 ms) was presented eight times by a room speaker to reduce habituation effects [7]. Participants were instructed to disregard the noise bursts during the experiment. Before and after the experiment, participants rated the acoustic startle probe on a 10-point scale (0 = not aversive, 10 = extremely aversive). The startle probe was rated as *M* = 5.81 (*SD* = 1.71) before the experiment, and its aversiveness decreased during the experiment significantly (*M* = = 5.03, *t*(61) = 2.70, *p* = 0.009, *d* = 0.34). The main experiment (Fig. 1B) started with a time-jittered white fixation cross (5500–7000 ms), followed by the presentation of a random picture (6000 ms), and a black screen (inter-stimulus interval, 11,000–13000 ms). Then, participants rated picture’s valence and arousal. The white noise burst was randomly applied to 49–50% of pictures in each condition, occurring either 2500 ms (25%) or 5500 ms (25%) after picture offset, and in 49% of the inter-stimulus intervals (randomized 500–1100 ms).

### Subjective ratings

Participants rated their subjective experience using a 9-point Likert scale using the SAM for valence (0 = pleasant to 8 = unpleasant) and arousal (0 = calm to 8 = arousing). Valence and arousal ratings were averaged across pictures for each category and participant.

### Recording system

Pictures were presented using Presentation Software (Version 18.0, Neurobehavioral Systems, Inc., Berkeley, CA). Psychophysiological measurements were recorded using a BioSemi ActiveTwo System (BioSemi, Amsterdam, Netherlands) at a sampling rate of 1024 Hz. The DRL electrode was placed over the ankle of the right leg, while the CMS electrode situated on the left forearm. The routine preprocessing pipeline of the laboratory was followed (see also [77]). The raw data was pre-processed and filtered using BrainVision Analyzer software (BrainVision Analyzer, Brain Products GmbH, Gilching, Germany). The filter type was identical for all measurements (24 dB/oct., zero-phase IIR Butterworth filter).

### Skin Conductance Response (SCR)

SCR was measured at the thenar and hypothenar of the non-dominant hand using Ag/AgCl electrodes (5-mm diameter, exosomatic measurement, isotonic electrolyte medium with 0.5% NaCl, 1 µA at 16 Hz AC). The data underwent offline low-pass filtering (1 Hz). Then, the SCR data was downsampled to 256 Hz and exported to Matlab. Using Ledalab [2], standard trough-to-peak SCR amplitudes (µS) were extracted per trial per participant in the time window between 1 to 6 s after stimulus onset,and exported for the statistical analysis.

### Heart Period (HP)

HP was recorded using a Lead II configuration (right arm, left leg) with AG/AGCl electrodes (4 mm diameter). The data underwent preprocessing and filtering with a bandpass filter (1 to 30 Hz) and a notch filter (50 Hz) in BrainVision Analyzer software. Following automated R-spike detection, the ECG data were manually inspected for artifacts, and a continuous HP trace was generated using Matlab R2019a (MathWorks, Natick, MA). The HP trace was segmented from −1000 to 5500 ms relative to picture onset and baseline-corrected (−1000 ms to 0 ms). HP was defined as the average changes in HP from 0 to 2 s and from 2 to 5 s after the presentation of the picture. These components are referred to as the A and D, respectively, representing the initial acceleration (HP-A) and subsequent deceleration (HP-D) in heart rate in response to emotional stimuli [14]. Mean HP activity (HP-A, HP-D) of all trials was exported for further analysis.

### Acoustic Startle Response (ASR)

Electromyographic activity was recorded using two Ag/AgCl electrodes (4 mm diameter) placed on the musculus orbicularis oculi. So, the startle itself was administered using a white noise burst (95 dB SPL, 500 ms), as most commonly used [114], displayed by a room speaker. The raw data was rectified and bandpass filtered with the range of 1 to 200 Hz. Then, the signal was smoothed using a 15 Hz low pass filter. Data was segmented from −100 to 250 ms based on category and baseline corrected (−100 to 0 ms). Further analysis was conducted using the analysis of single trials, thus maximizing the individual response peaks. The maximum peak was identified using the peak detection algorithm integrated into BrainVision Analyzer. Startle amplitude was defined as the maximum peak amplitude within the time interval of 50 and 200 ms following the onset of the white noise burst. If more than one peak was detected, the maximum peak amplitude was still chosen, as long as there was no other discernible response [7]. All trials were exported and z-standardized before the multilevel analysis. The number of ASR differed per category. In SIO, only six ASRs were measured, possibly limiting their reliability.

### Power analysis

The power calculation was performed using G*Power [28]). Assuming a medium effect size (*f*^*2*^ = 0.2), a minimum of 58 participants is required to achieve a power of 0.80 for a two-sided test.

### Statistical analysis

Intraclass correlations were computed to assess the interrater reliability using SPSS (IBM SPSS Statistics for Windows, [97], Armonk, NY: IBM Corp). Following Koo and Li [52], intra-class correlation coefficients (consistency type) were calculated for each category (Neutral Objects, Self-Injury Objects, Scenes with Self-Injury) for valence and arousal ratings, employing a two-way random effects model with the mean of the 64 participants.

Using RStudio [90], multilevel analyses were conducted with the *nlme* package [79], and post-hoc power analyses were performed with the *simr* package [36]. The *emmeans* package [59] was used to compute contrasts between picture categories, applying Tukey *p*-value adjustment and Kenward-Roger degrees of freedom.

For each dependent variable (Valence, Arousal, Heart Period Component A, Heart Period Component D, Acoustic Evoked Startle Response, Skin Conductance Response), multilevel-models were fitted. Fixed effects included picture Category; Participant ID was modelled as a random effect to account for individual differences. Trials (Trial 1 to Trial 89) were nested as repeated measures within Participant ID.

To examine the effects of time/habituation (Trial) and Startle (whether a startle was applied in the trial: yes/no), both variables were included as fixed factors in each model [84, 104]. In the ASR model specifically, instead of Startle, the variable Startle Segments (Segments 1 to 44) was included to represent the successive timing of startle presentation.

To assess whether pre- and post-self-injury pictures significantly impacted physiological variables, a dummy-coded variable (Post-Self-Injury: 0 = pre-self-injury, 1 = post-self-injury) was added. Four models were estimated: (1) Nullmodel: a null model without any predictors; (2) M1: a random intercept model with Category, Trial, and Startle as fixed effects and ID as random intercept; (3) M2: a random intercept model allowing interactions between Category and Trial (to capture different habituation gradients per Category), and between Category and Startle (to account for different startle response depending on Category); (3) M3: a random intercept model with Category as a random slope; (4) M4: a random intercept/random slope model including Post-Self-Injury as a predictor.

When the model M3 was singular, the random slope was not included in M4. The final model selection was based on fit indices (AIC/BIC) and convergence criteria, with singular models excluded. Specifically, M4 was reported for Valence and Arousal; M3 was reported for SCR; M2 was reported for HP-A, HP-D, and ASR.^2^ All tables summarizing models are provided in the Supplementary Materials. Significance levels were reported two-tailed. All R scripts, stimuli, and data used in the analysis have been published online (see https://osf.io/hpswr/).

## Results

### EPSI validation

**Fig. 2:**
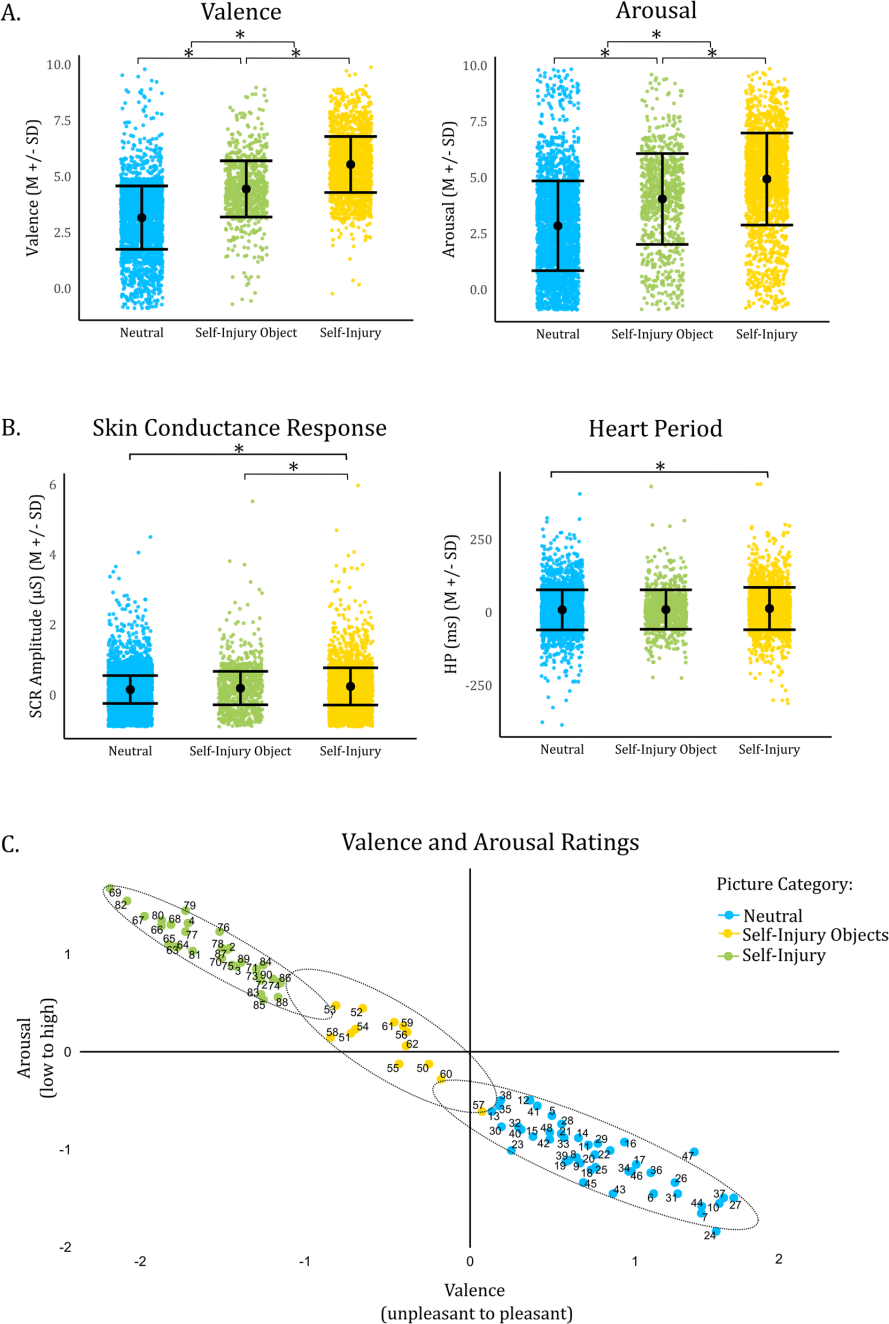
**A** Scatter Plot of Valence and Arousal Ratings (Mean and Standard Deviation). Participants significantly differentiated between all categories (*** significant at *α* <.001). **B** Mean and SD of HP and SCR. SCR response was significantly enhanced in scenes with self-injury compared to neutral objects (*** significant *α* <.001) and images depicting self-injury object (** significant at *α* <.01). HP was significantly increased in images depicting self-injury compared to neutral objects. **C** Valence and Arousal Ratings of every picture. Categories are colored, with ellipses marking the variances, and scales were transformed into a bipolar scale ranging from pleasant to unpleasant and low arousal to high arousal

The mean valence was *M* = 3.12 (*SD* = 0.89, [Min–Max: 0.67, 6.44]) for NO, *M* = 4.44 (*SD* = 0.72, [2.10, 6.10]) for SIO and *M* = 0.54 (*SD* = 0.92, [3.68,5.54]) for SSI. The mean arousal was *M* = 2.85 (*SD* = 1.59, [0.00, 8.74]) for NO, *M* = 4.05 (*SD* = 1.68, [0.00, 9.00]) for SIO, and *M* = 4.94 (*SD* = 1.72, [0.65, 4.94]) for SSI (see Fig. 2A). The mean item-total correlation of valence ratings was *r* = 0.68 (SD = 0.11, [0.27, 0.89]) for NO, *r* = 0.75 (SD = 0.08 [0.53, 0.89]) and *r* = 0.60 (SD = 0.09, [0.42, 0.72]) for SSI. The mean item-total correlation of arousal ratings was *r* = 0.82 (SD = 0.06, [0.55, 0.93]) for NO, *r* = 0.84 (SD = 0.06, [0.71, 0.93]), and *r* = 0.83 (SD = 0.05, [0.77, 0.91] for SSI, indicating acceptable to high reliability.

The ICC (2, 64) for valence ratings was 0.91 [95% CI: 0.87, 0.94.] for NO, 0.81 [0.63, 0.93] for SIO and 0.86 [0.77, 0.92] for SSI. For arousal ratings, the ICC (2, 64) was 0.80 [0.70, 0.87] for NO, 0.76 [0.53, 0.91] for SIO, and 0.72 [0.57, 0.85] for SSI. When combining SIO and SSI into one category, the ICC increased to 0.96 [0.93, 0.97] for valence and 0.91 [0.586, 0.94] for arousal ratings. This indicates good to excellent agreement [52].

The mean valence ratings of NO were significantly correlated (*r* = 0.53) with the mean valence ratings of SIO; but not associated with the valence ratings of SSI (*r* = −0.02), indicating good differentiation for SSI. The mean arousal ratings showed moderate to high associations (*r* = 0.64 to *r* = 0.84) across picture categories. The arousal ratings of NO were less correlated with the arousal ratings of SSI (*r* = 0.64) than with SIO (*r* = 0.84). Valence ratings of NO were only moderately associated with arousal ratings of SIO (*r* = 0.26) and not correlated with SSI (*r* = 0.11), indicating a clear differentiation between the arousal and valence dimensions of NO versus SIO and SSI (see Supplement).

### Valence ratings (M4)

Significant effects were observed for Self-Injury Objects (*b* = 1.57, SE = 0.13, *t*(188.26) = 12.05, *p* < 0.001), Self-Injury (*b* = 2.28, SE = 0.17, *t*(86.46) = 13.18, *p* < 0.001), Trial (*b* = 0.00, SE = 0.00, *t*(5501.73) = 2.06, *p* = 0.04), Startle (*b* = 0.13, SE = 0.04, *t*(5497.31) = 3.34, *p* < 0.001), Post-Self-Injury (*b* = 0.46, SE = 0.05, *t*(5497.07) = 9.74, *p* < 0.001), and the interaction effects of Category and Trial (SIO × Trial: *b* = 0.00, SE = 0.00, *t*(5454.17) = −2.82, *p* < 0.001; SSI × Trial: *b* = 0.00, SE = 0.00, *t*(5507.09) = −2.55, *p* = 0.01).

Contrasts revealed significantly higher valence ratings in SIO compared to NO (*t*_NO-SIO_ (63) = −13.02, *p* < 0.001), SSI compared to NO (*t*_NO-SSI_ (66.7) = −13.01, *p* < 0.001) and SSI compared to SIO (*t*_SIO-SSI_ (70.7) = −7.17, *p* < 0.001). The effect of Trial suggested that valence ratings decreased over time during the experiment—potentially reflecting habituation effects where repeated presentation of similar stimuli reduces perceived unpleasantness/aversiveness in affective judgments—especially pronounced in SSI and SIO images that were initially rated as more aversive. The effect of Startle indicated that pictures paired with a startle were perceived as more aversive across categories. Additionally, post-self-injury pictures also appeared more unpleasant (*b* = 0.46, SE = 0.05, *t*(5497.07) = 9.75, *p* < 0.001).

### Arousal ratings (M4)

Significant effect were found for SIO (*b* = 1.46, SE = 0.16, *t*(172.11) = 9.29, *p* < 0.001), SSI (*b* = 2.13, SE = 0.19, *t*(89.24) = 11.03, *p* < 0.001), Startle (*b* = 0.34, SE = 0.04, *t*(5497.25) = 7.71, *p* < 0.001), and Post-Self Injury (*b* = 0.39, SE = 0.05, *t*(5497.04) = 7.18, *p* < 0.001). The effect of Trial was not significant, but the effect of Startle (*b* = 0.34, SE = 0.04, *t*(5497.25) = 7.71, *p* < 0.001). Contrasts confirmed increased arousal in SIO vs. NO (*t*_NO-SIO_ (63) = −9.86, *p* < 0.001), SSI vs. NO (*t*_NO-SSI_ (67.1) = −10.35, *p* < 0.001), and SSI vs. SIO (*t*_SIO-SSI_ (71.8) = −5.27, *p* < 0.001). Pictures associated with the startle were generally rated as more arousing, similarly to post-self-injury pictures. The interaction effects of SIO/SSI × Startle were significant (*b* = −0.21/−0.22, SE = 0.09/0.07, *t*(5507.62/5497.12) = −2.21/−3.21, *p* = 0.03/< 0.001), possibly indicating a misattribution of arousal toward the startle rather than the specific image content.

### SCR. (M3)

The fixed effect of SSI showed a significant effect on SCR (*b* = 0.18, SE = 0.03, *t*(362.88) = 5.58, *p* < 0.001; Fig. 2B). The effect of Trial was also significant (b = 0.00, SE = 0.00, *t*(5516.97) = −6.29, *p* < 0.001), indicating an overall decrease in SCR across trials. The effect of Startle (*b* = 0.14, SE = 0.02, *t*(5496.44) = 8.94, *p* < 0.001) was significant as well, suggesting increased SCR to pictures presented with the startle. The interaction between SSI and Trial was significant (*b* = 0.00, SE = 0.00, *t*(5514.71) = −4.08, *p* < 0.001), indicating that SCR changes over trials differed depending on the category. Specifically, a habituation effect where SCR decreased over trials was especially prominent in the SSI category, likely because these images elicited higher SCR initially. Contrasts revealed increased SCR in SSI compared to NO (*t*_NO-SSI_ (62.8) = −4.73, *p* < 0.001), and SSI compared to SIO (*t*_SIO-SSI_ (62.6) = −2.91, *p* = 0.014). The comparison between SIO to NO approached significance (*t*_NO-SIO_ (62.7) = −2.25, *p* = 0.07).

### HP. (M2)

No significant effects of Category emerged in the HP-A model. However, trial was significantly associated with HP (*b* = −0.27, SE = 0.07, *t*(5623.93) = −3.99, *p* < 0.001), indicating a decrease in HP over time. In the HP-D model (M2), significant effects were observed for SSI (*b* = 15.66, SE = 4.45, *t*(5625.19) = 3.52, *p* < 0.001), Trial (*b* = −0.17, SE = 0.05, *t*(5623.72) = −3.58, *p* < 0.001), and the interaction effect between SSI and Trial (*b* = −0.25, SE = 0.08, *t*(5630.89) = −3.18, *p* < 0.001). A direct comparison showed that SSI had increased HP compared to NO (*t*_NO-SSI_ (5615) = −2.42, *p* = 0.04). The effect of Trial indicated a reduction in HP (specifically, less deceleration), as the number of trials increases. This effect was especially pronounced in the SSI category.

### ASR. (M2)

No significant effect of Category was found in M2 for the ASR. However, the effect of Startle Segments was significant (*b* = −0.02, SE = 0.01, *t*(650.80) = −3.12, *p* < 0.001), indicating a decrease in startle amplitude over time.

## Discussion

This pilot study offers a first validation of the EPSI, which includes pictures of neutral objects, objects related to self-injury, and scenes depicting self-injury, in a healthy sample. Pictures were presented in random order in a picture viewing paradigm while assessing subjective ratings of valence and arousal and physiological markers of emotional processing (SCR, HP, ASR). The development of the EPSI aimed to create a standardized picture set that not only includes images depicting self-injury and self-injury objects but also facilitates research into the NSSI process itself by incorporating different stages of NSSI.

Regarding reliability, intraclass correlation was moderate to high across all categories for both valence and arousal ratings. Similarly, the item-total correlation demonstrated consistently high reliability across all categories. All in all, these results demonstrate sufficient reliability along the item-statistical dimensions. Regarding validity, participants reported significantly higher negative valence and arousal in SSI compared to SIO and NO. Additionally, SIO were rated as significantly more aversive and arousing than NO. There was no correlation between SSI images and the valence and arousal ratings of NO. Furthermore, participants successfully distinguished between the pre- and post-self-injury stages in their self-reports of arousal and valence.

At the physiological level, the SCR significantly increased in SSI compared to SIO and NO, while HP showed a trend towards significance. However, no differences were found in the ASR. No significant differences were found between stages of self-injury in all physiological measures. In summary, while self-reports clearly support the validity and reliability of EPSI, physiological results partially support these findings. SCR emerged as the most robust physiological marker.

These results are in line with previous research on the processing of emotional pictures. In the context of IAPS, a boomerang-shaped pattern of valence and arousal has been reported [18]. Thus, the results replicated previous studies indicating increased arousal and negative valence when processing aversive content [14]. Since EPSI did not contain ‘positive’ pictures, the pattern was partially replicated. Conceptually, pictures containing aversive content activate the emotional defense system, leading to increased attentional engagement (as mirrored in HP deceleration), increased sympathetic activation (increased SCR), while inducing a pronounced startle response as marker of attentional engagement [63]. On the subjective level, this psychological state is mirrored by increased valence and arousal ratings [12]. As participants rated the EPSI categories as significantly different, categories are indeed distinct regarding subjective emotional processing and valid of eliciting specific emotional reactions.

SCR has been associated with physiological arousal, indicating sympathetic autonomic activation [26, 93]. It increases with the subjective emotional arousal, reflecting the level of motivational activation of the emotional defense system [9, 55, 56] and has been observed to be elevated in response to physical harm and mutilation [9, 14]. Consistently, the SCR did differ between SSI and NO, but not between NO and SIO. Possibly, the presented self-harm objects (e.g., household items like iron) are not perceived as threatening by healthy participants. Indeed, Plener et al. [80] reported altered processing of SSI in patients compared to healthy participants. This explanation aligns with the fact that no physiological differences between stages of self-injury emerged. As patients report decreasing arousal during self-injury [49], further studies could explore whether different stages of NSSI indeed lead to different activations of the defense system in a clinical, but not healthy sample. The EPSI pictures may provide insight into the timeline of NSSI-associated emotional defense systems and, at the level of NSSI stages, into the physiological factors involved in maintaining the disorder [71, 86].

Unpleasant or threatening stimuli elicit a HP deceleration [9, 34], indicating an initial reaction of the defensive cascade to potentially harmful stimuli. It signifies sustained attention without immediate action, and is primarily mediated by the parasympathetic nervous system [56]. In the present study, HP slightly differed between SSI and NO, but not between the other categories. Even though this effect was small, SSI might elicit an increased defensive engagement.

Changes in the ASR were not significantly associated with any picture category. The motor response of the startle reflex is inhibited in moderately arousing, unpleasant contexts to focus attention and gather information, while in states of increasing threat, it is primed for defensive action [9, 11, 16]. In line with SCR and HP, the pictures might have been moderate, but not highly unpleasant and arousing, thus rather inhibiting than activating the defense system. Hence, they may have induced a state of information gathering. However, ASR number was low, even though Lieberman et al. [60] reported a minimum of six ASR necessary for reliability.

The present pilot study has several limitations. First, the sample used in the study was neither representative nor did it include a clinical sample. In general, representativeness is a highly desired outcome, which is unfortunately rarely achieved in empirical studies, especially in the field of psychophysiology, which suffers from chronically small sample sizes due to its complex experimental procedures [57]. In the context of the present study, this limitation restricts the generalization of the findings to a population of students aged 18 to 30. Furthermore, the current sample consists predominantly of young women, potentially introducing a gender and age bias. While previous studies did not report gender differences in valence and arousal self-reports [4, 111], psychophysiological measures are indeed prone to age effects [66]. Considering that NSSI in clinical samples peaks between ages 20 to 24 [31], the present sample could be considered close to a population sample. This highlights another limitation of the present pilot study: While the presence of thoughts of self-injury among participants was accounted for, prior exposure to NSSI was not– an influence that may arise through contact with peers in engaging in NSSI. Such exposure may serve as a risk factor in the development of the behavior itself [105], and, in the context of the present study, may have contributed to decreased sensitivity towards the self-injury stimuli. Future studies could not only strive for a representative sample but also expand their scope to include indirect exposure to NSSI, even when participants themselves are not engaging in it.

No clinical sample was examined. This leads to significant drawbacks when interpreting the psychophysiological reactions of the participants, as it remains unclear whether these reactions are specific to individuals with or without NSSI. Since this study served as a first evaluation of the EPSI, a psychiatric population was not included in the sample. As suggested in the protocol of our validation study [1], a clinical study to validate the use of the EPSI in a sample of patients with borderline personality disorder is in planning. At the same time, recent studies suggest that altered attentional processes in NSSI play a more significant role than parasympathetic and sympathetic mediated emotional responses to aversive stimuli [32, 33]. Furthermore, there is tentative evidence that contextual factors, such as stress, influence the reactivity of individuals engaging in NSSI to emotional stimuli [100]. This, in turn, shifts the focus toward the process of NSSI and its situational moderators. These elements can be analyzed physiologically, emotionally, and cognitively using the EPSI, which segments the behavior along a timeline of pre- and post-self-injury stages. So far, the present pilot study provides preliminary evidence that the stimuli of the EPSI and its categories elicit distinct emotional responses in healthy participants.

Furthermore, as the present study offers a preliminary evaluation of *all* pictures in the EPSI, the images were not compared to those depicting non-self-inflicted injury or blood, such those found in the IAPS. This makes it difficult to determine whether emotional and physiological reactivity is a result of the NSSI or exposure to blood or injury [9, 13]. Although the present study did not include IAPS pictures of accidents and injuries, images containing self-injury content without blood were part of the study. The pictures depicting self-injury in its initial stages (pre-self-injury) did not show any signs of blood or injury. While later stages of self-injury were rated as less pleasant and more arousing in subjective self-reports, psychophysiological reactivity measured by SCR and heart rate did not differ significantly between the pre- and post-self-injury stages. Tentatively, this may indicate that physiological responses, particularly in SCR, might be a result of the NSSI rather than the display of blood and injury. Nevertheless, further studies should include comparable images to determine whether the emotional reactivity arises is from scenes involving injury or blood versus NSSI.

At the same time, including all pictures in the pilot study led to the introduction of stimulus imbalance at the category level. While the neutral object category contained 45 pictures, the SSI category included only 31 pictures, followed by 13 pictures in the SIO category. This may result in considerable differences in variance and standard deviation, possibly leading to biased item statistics and a general reduction in statistical power, particularly for the SIO category. However, regarding reliability measures and physiological variables, SIO pictures did not show significantly higher variance or standard deviation compared to SSI and NO. Furthermore, post-hoc power analyses indicated sufficient statistical power for all main results (see Supplement).

Finally, the last point: The physiological results were less pronounced as expected, with the SCR primarily indicating that physiological arousal is the driving factor behind the differences in emotional responses to the NSSI. Similar emotional processes have been primarily studied in the IAPS, which differs in content and picture intensity. EPSI was developed to specifically target NSSI rather than emotions in general, limiting the variety of picture content. Furthermore, it explicitly depicts different stages of self-injury, resulting in a certain degree of redundancy. Its redundancy could lead to habituation (as reported only for the physiological variables), which is a common challenge in picture viewing paradigms [16]. On top, a healthy sample displays reduced emotional reactivity as result of stimulus relevance. NSSI patients would likely rate SSI and SIO differently, as these stimuli are highly relevant to the disorder, and a dysregulation of the emotional arousal system is hypothesized to underlie the disorder [73]. However, further studies should directly compare clinical samples to investigate the role of arousal in emotional reactivity to the EPSI pictures.

The EPSI aimed to depict various ways of self-injury. However, not all potential types of NSSI are represented, even though it contains the most common forms, and many individuals engage in multiple forms [21, 35, 58, 109].

## Conclusions

The long-term goal of developing the EPSI was to create a standardized stimulus set, accessible for any research in the field, that elicits emotional responses to NSSI stimuli in healthy and clinical samples. Most importantly, the EPSI allows for the exploration of different stages by segmenting the timeline of NSSI.

The present study is the first to explore its utility in eliciting distinct emotional reactivity in a healthy sample. This pilot study provides initial evidence that the EPSI may be a valid measure of distinct emotional processing, particularly on the level of self-reports. Psychophysiological reactivity, as indicated by arousal (measured via SCR), partially supports these results. Further studies should include comparable images depicting non-self-inflicted injuries, include a clinical sample to test its clinical validity, and aim for representativeness. In the long term, the clinical application of the EPSI as a potential measure of therapeutic success remains to be examined.

## Supplementary Information

Supplementary Material 1.

Supplementary Material 2.

## Data Availability

No datasets were generated or analysed during the current study.

## Footnotes

1 To this end, neutral pictures were re-used from a picture set developed for eating disorders, see Blechert et al. [5]. SSI and SIO pictures of the EPSI are available online.

2 Note that adding the random component affects the estimated means and, consequently, the test statistic, resulting in a more conservative estimate of the effect when heteroscedasticity is present.
