# Supplementary material for: First evaluation of the emotional picture set of self-injury images (EPSI) using psychophysiological and self-report measures
Source: Borderline Personal Disord Emot Dysregul. 2025 Jul 12;12:27. doi: 10.1186/s40479-025-00304-4 (PMC12255983; doi:10.1186/s40479-025-00304-4)
Supplement: Supplementary file 1 — Supplementary Material 1. [file 40479_2025_304_MOESM1_ESM.docx]

**Supplement**

*Table 1.* Demographic information and questionnaires

|  | **All**  *N* = 64 | **Female**  *n* = 46 | **Male**  *n* = 18 |
| --- | --- | --- | --- |
| **Age** (*M*, *SD*)^1^ | 22.41 (3.12) | 21.78 (3.0) | 24.00 (3.10) |
| **Height** (*M*, *SD*)^1^ | 172.20 (9.1) | 168.37 (6.90) | 181.89 (6.40) |
| **Weight** (*M*, *SD*)^1^ | 67.34 (13.92) | 62.67 (10.91) | 79.28 (13.89) |
| **BMI** (*M*, *SD*)^1^ | 22.62 (3.72) | 22.07 (3.39) | 23.98 (4.24) |
| **No Smoking** (%, *n*)^2^ | 74.60% (47)^1^ | 80.4 % (37)^8^ | 55.6 % (10) |
| **No hormonal birth control** (%, *n*)^2^ | 79.69 % (51) | 71.7 % (33) | / |
| **No Previous Drug Use** (%, *n*)^2^ | 69.84% (44)^1^ | 82.6 % (38)^8^ | 33.3% (6) |
| **NEO-FFI**^4^ (*M*, *SD*)^1^  Neuroticism  Extraversion  Openness  Conscientiousness  Agreeableness | 1.97 (.57)  2.56 (.62)  2.47 (.54)  2.76 (.75)  2.87 (.35) | 2.00 (.46)  2.61 (.59)  2.50 (.52)  2.85 (.74)  2.91 (.34) | 1.90 (.81)  2.44 (.72)  2.39 (.59)  2.55 (.73)  2.77 (.35) |
| **BSL-23**^5^ (*M*, *SD*)^1^ | 1.57 (.42) | 1.55 (.40) | 1.67 (.48) |
| **PANAS**^6^ (*M*, *SD*)^1^  Positive Affect  Negative Affect | 2.23 (.47)  2.50 (.49) | 2.24 (.44)  2.55 (.47) | 2.19 (.55)  2.37 (.53) |
| **SPF-IRI**^7^ (*M_Sum_*, *SD*)^3^  Fantasy  Empathic Concern  Perspective-Taking  Personal Distress | 12.92 (3.29)  13.33 (3.10)  14.25 (3.74)  10.09 (2.36) | 13.17 (3.29)  13.57 (3.14)  14.40 (3.81)  10.20 (2.36) | 12.28 (3.30)  12.72 (2.80)  13.89 (3.66)  9.83 (2.38) |

^1^ Body Mass Index, Mean, Standard Deviation; ^2^ Percentage, Number; ^3^ Mean Sum Score, Standard Deviation; ^4^ NEO-Five-Factor Inventory (Kanning, 2009); ^5^ Borderline-Symptom Checklist (BSL-23; Bohus et al., 2001); ^6^ Positive and Negative Affect Scale (PANAS-SF; Breyer & Bluemke, 2016; Watson et al., 1988); ^7^ Saarbrücker Persönlichkeitsfragebogen Empathie (Paulus, 2009); ^8^ Missing: 1.56 % (1).

*Table 2*. Correlations (Pearson’s *r*) of valence and arousal ratings per category (N = 64)

|  |  | **Correlation (Pearson’s *r*)** | | | | | | | | | | |
| --- | --- | --- | --- | --- | --- | --- | --- | --- | --- | --- | --- | --- |
|  |  | **Valence**^1^ | | | | |  | **Arousal**^1^ | | | | |
|  |  | NO |  | SIO |  | SSI |  | NO |  | SIO |  | SSI |
| **Valence** |  |  |  |  |  |  |  |  |  |  |  |  |
| NO |  | 1 |  | .50** |  | -0.03 |  | .60** |  | .28* |  | .10 |
| SIO |  |  |  | 1 |  | .50** |  | .50** | - | .64** |  | .49** |
| SSI |  |  |  |  |  | 1 |  | .12 |  | .32** |  | .56** |
| **Arousal** |  |  |  |  |  |  |  |  |  |  |  |  |
| NO |  |  |  |  |  |  |  | 1 |  | .78** |  | .58** |
| SIO |  |  |  |  |  |  |  |  |  | 1 |  | .83** |
| SSI |  |  |  |  |  |  |  |  |  |  |  | 1 |

* Significant at *α* < .05, **significant at *α* < .01. ^1^ Valence and Arousal Ratings as measured by the Self-Assessment Manikin (SAM; Bradley & Lang, 1994).

*
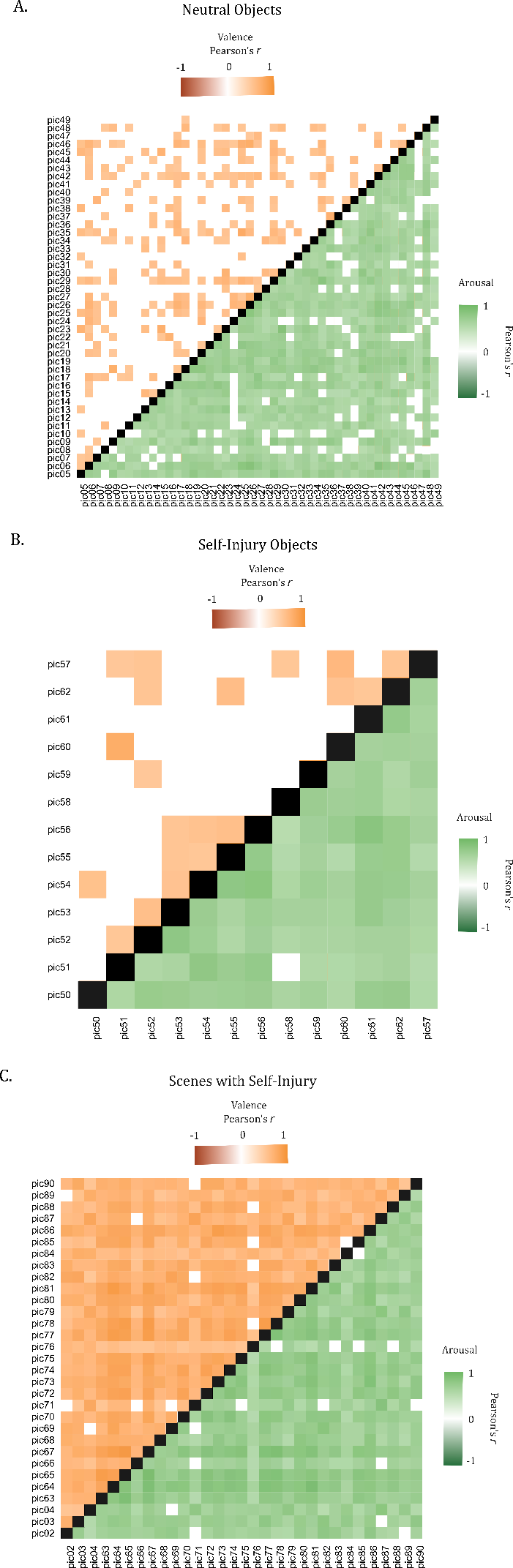
F**igure 1*. **A.** Pearson’s correlation (*r* > .50) between pictures depicting neutral objects.Valence dimension is colored in orange, while the arousal dimension is colored in green. The auto-correlation is colored in black. At the arousal dimension, most pictures are highly positively correlated. **B.** Pearson’s correlation (*r* > .50) between pictures depicting scences with self-injury objects. Valence dimension is colored in orange, while the arousal dimension is colored in green. The auto-correlation is colored in black. At the arousal dimension, most pictures are highly positively correlated. **C.** Pearson’s correlation (*r* > .50) between pictures depicting scences with self-injury. Valence dimension is colored in orange, while the arousal dimension is colored in green. The auto-correlation is colored in black. At the valence and arousal dimension, most pictures are highly positively correlated.

*
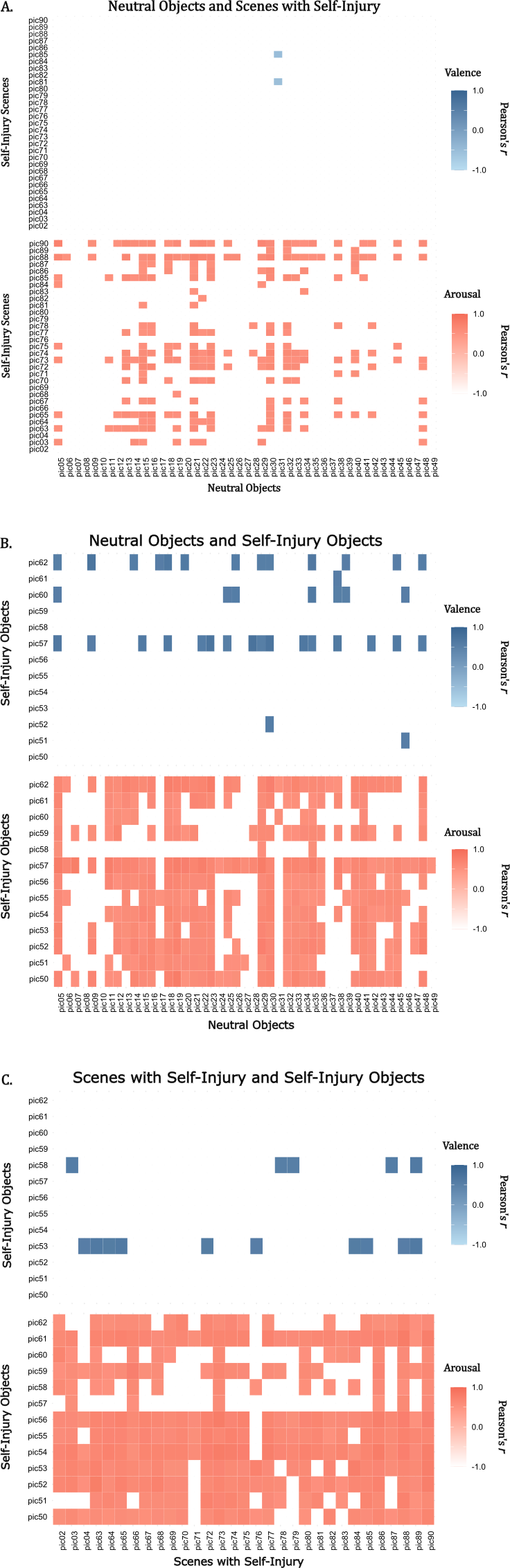
Figure 2.* **A.** Pearson’s correlation (*r* > .50) between pictures depicting neutral objects and scenes with self-injury. The valence dimension is colored in blue, while the arousal dimension is colored in red. Most pictures are not highly associated **B.** Pearson’s correlation (*r* > .50) between pictures depicting neutral objects and self-injury objects. Valence dimension is colored in blue, while the arousal dimension is colored in red. **C.** Pearson’s correlation (*r* > .50) between pictures depicting scences with self-injury and self-injury objects. Valence dimension is colored in blue, while the arousal dimension is colored in red.

*
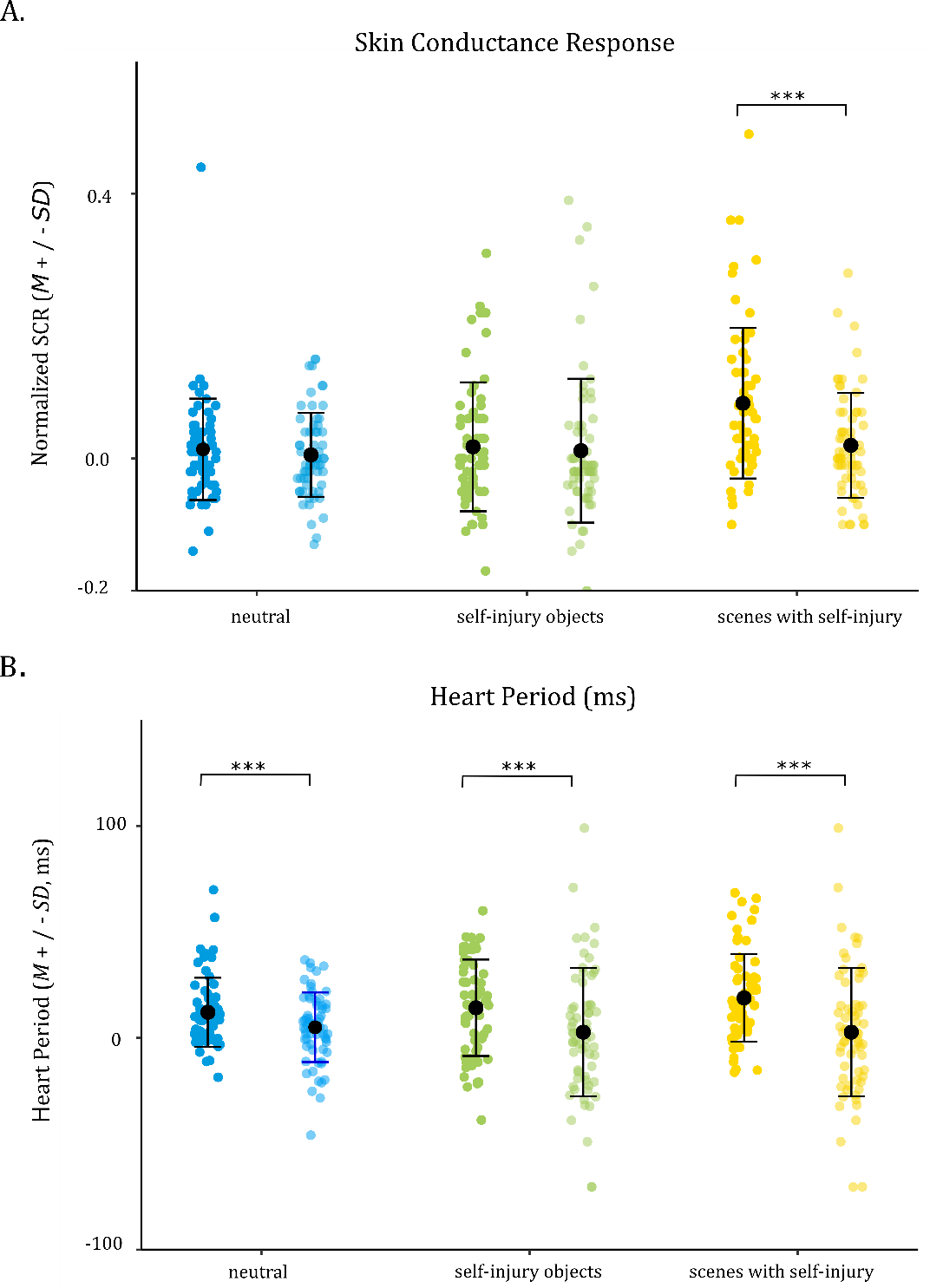
*

*Figure 3.* **A**. Scatter Plot of temporal effects per category of SCR (*** significant at *α* < .01). Temporal effects were tested using split-half method, resulting in the mean of the first half (T1) and the mean of the second half (T2) of the trials. The mean of the second halves were marked in lighter colour. Self-injury pictures showed significant differences between the first and the last half of trials. **B**. **A**. Scatter Plot of temporal effects per category of HP (** significant at *α* < .01). Temporal effects were tested using split-half method, resulting in the mean of the first half (T1) and the mean of the second half (T2) of the trials. The mean of the second halves were marked in lighter colour. All categories displayed significant differences between trials shown in the first half (T1) and those shown in the second half (T2).

*Table 3*. Valence and arousal ratings of the EPSI (mean and standard deviation)

| **Picture Category** | **Valence** | | | **Arousal** | | |
| --- | --- | --- | --- | --- | --- | --- |
|  | *M* | *SD* | | *M* | *SD* | |
| SI | 5.48 | 1.28 | | 5.00 | 2.02 | |
| SI | 5.52 | 1.35 | | 4.94 | 1.98 | |
| SI | 5.78 | 1.28 | | 5.25 | 1.79 | |
| N | 3.56 | 1.25 | | 3.36 | 1.86 | |
| N | 2.89 | 1.35 | | 2.53 | 1.92 | |
| N | 2.56 | 1.77 | | 2.38 | 1.95 | |
| N | 3.33 | 1.20 | | 2.86 | 1.65 | |
| N | 3.38 | 1.68 | | 2.84 | 2.03 | |
| N | 2.52 | 1.25 | | 2.41 | 1.89 | |
| N | 3.23 | 1.22 | | 3.00 | 1.85 | |
| N | 3.63 | 1.29 | | 3.44 | 2.05 | |
| N | 3.88 | 1.06 | | 3.41 | 1.80 | |
| N | 3.33 | 1.35 | | 3.13 | 1.79 | |
| N | 3.66 | 1.00 | | 3.08 | 1.80 | |
| N | 3.05 | 1.40 | | 3.05 | 1.89 | |
| N | 2.95 | 1.56 | | 2.78 | 2.04 | |
| N | 3.25 | 1.26 | | 2.81 | 1.85 | |
| N | 3.41 | 1.48 | | 2.91 | 1.89 | |
| N | 3.14 | 1.60 | | 2.94 | 2.08 | |
| N | 3.48 | 1.38 | | 3.14 | 1.95 | |
| N | 3.17 | 1.23 | | 3.02 | 1.84 | |
| N | 3.77 | 0.99 | | 2.98 | 1.73 | |
| N | 2.42 | 1.62 | | 2.08 | 2.02 | |
| N | 3.22 | 1.31 | | 2.83 | 2.09 | |
| N | 2.81 | 1.56 | | 2.67 | 2.08 | |
| **Image Category** | **Valence** | | | **Arousal** | | |
|  | *M* | *SD* | | *M* | *SD* | |
| N | 2.36 | 1.30 | | 2.39 | 2.01 | |
| N | 3.47 | 1.32 | | 3.20 | 1.98 | |
| N | 3.27 | 1.35 | | 3.03 | 2.04 | |
| N | 3.78 | 1.09 | | 3.27 | 2.07 | |
| N | 2.69 | 1.39 | | 2.50 | 1.90 | |
| N | 3.70 | 1.14 | | 3.27 | 1.81 | |
| N | 3.42 | 1.22 | | 3.06 | 1.78 | |
| N | 3.08 | 1.61 | | 2.81 | 1.97 | |
| N | 3.88 | 1.34 | | 3.55 | 2.12 | |
| N | 2.92 | 1.49 | | 2.78 | 1.96 | |
| N | 2.47 | 1.47 | | 2.48 | 2.02 | |
| N | 3.78 | 1.09 | | 3.45 | 1.96 | |
| N | 3.39 | 1.41 | | 2.89 | 1.74 | |
| N | 3.67 | 1.01 | | 3.23 | 1.93 | |
| N | 3.56 | 1.32 | | 3.47 | 1.98 | |
| N | 3.42 | 1.21 | | 3.03 | 2.01 | |
| N | 3.14 | 1.13 | | 2.53 | 1.82 | |
| N | 2.69 | 1.42 | | 2.47 | 1.95 | |
| N | 3.31 | 1.34 | | 2.69 | 1.96 | |
| N | 3.00 | 1.47 | | 2.70 | 1.89 | |
| N | 2.64 | 1.57 | | 3.02 | 2.13 | |
| N | 3.53 | 1.17 | | 3.16 | 1.84 | |
| N | 2.08 | 1.35 | | 2.44 | 1.83 | |
| SIO | 4.25 | 1.05 | | 3.88 | 1.97 | |
| SIO | 4.73 | 1.16 | | 4.19 | 1.97 | |
| SIO | 4.69 | 1.26 | | 4.44 | 2.00 | |
| SIO | 4.91 | 1.24 | | 4.53 | 1.98 | |
| **Image Category** | **Valence** | | | **Arousal** | | |
|  | *M* | | *SD* | *M* | | *SD* |
| SIO | 4.80 | 1.17 | | 4.31 | 1.91 | |
| SIO | 4.52 | 1.23 | | 3.95 | 1.96 | |
| SIO | 4.41 | 0.97 | | 4.22 | 1.87 | |
| SIO | 3.95 | 1.12 | | 3.41 | 1.86 | |
| SIO | 4.89 | 1.61 | | 4.13 | 1.87 | |
| SIO | 4.38 | 1.44 | | 4.27 | 2.02 | |
| SIO | 4.19 | 1.01 | | 3.78 | 1.84 | |
| SIO | 4.48 | 1.37 | | 4.30 | 1.99 | |
| SIO | 4.47 | 1.43 | | 4.16 | 2.02 | |
| SI | 5.83 | 1.39 | | 5.06 | 2.08 | |
| SI | 5.89 | 1.33 | | 5.08 | 1.99 | |
| SI | 5.88 | 1.25 | | 5.02 | 2.13 | |
| SI | 5.94 | 1.27 | | 5.36 | 1.97 | |
| SI | 6.02 | 1.27 | | 5.39 | 2.24 | |
| SI | 5.84 | 1.29 | | 5.22 | 1.85 | |
| SI | 6.19 | 1.41 | | 5.63 | 2.00 | |
| SI | 5.61 | 1.33 | | 5.02 | 1.94 | |
| SI | 5.31 | 1.13 | | 4.83 | 1.84 | |
| SI | 5.31 | 1.17 | | 4.67 | 1.99 | |
| SI | 5.34 | 1.24 | | 4.78 | 1.83 | |
| SI | 5.20 | 1.16 | | 4.66 | 2.03 | |
| SI | 5.47 | 1.11 | | 4.89 | 1.89 | |
| SI | 5.58 | 1.26 | | 5.27 | 1.74 | |
| SI | 5.77 | 1.21 | | 5.19 | 1.93 | |
| SI | 5.63 | 1.25 | | 5.02 | 2.07 | |
| SI | 5.77 | 1.31 | | 5.45 | 2.02 | |
| SI | 5.92 | 1.13 | | 5.34 | 2.03 | |
| **Image Category** | **Valence** | | | **Arousal** | | |
|  | *M* | *SD* | | *M* | *SD* | |
| SI | 5.73 | 1.36 | | 4.97 | 2.10 | |
| SI | 6.14 | 1.41 | | 5.52 | 1.99 | |
| SI | 5.36 | 1.20 | | 4.63 | 2.00 | |
| SI | 5.31 | 1.22 | | 4.89 | 1.87 | |
| SI | 5.23 | 1.19 | | 4.56 | 1.98 | |
| SI | 5.23 | 1.15 | | 4.67 | 1.93 | |
| SI | 5.50 | 1.25 | | 5.11 | 2.00 | |
| SI | 5.23 | 1.15 | | 4.63 | 1.80 | |
| SI | 5.50 | 1.28 | | 5.05 | 2.13 | |
| SI | 5.25 | 1.21 | | 4.81 | 1.88 | |
