# Supplementary material for: First evaluation of the emotional picture set of self-injury images (EPSI) using psychophysiological and self-report measures
Source: Borderline Personal Disord Emot Dysregul. 2025 Jul 12;12:27. doi: 10.1186/s40479-025-00304-4 (PMC12255983; doi:10.1186/s40479-025-00304-4)
Supplement: Supplementary file 2 — Supplementary Material 2. [file 40479_2025_304_MOESM2_ESM.docx]

**Supplement**

**Table 1***.* Demographic Information and Questionnaires

|  | **All**  *N* = 64 | **Female**  *n* = 46 | **Male**  *n* = 18 |
| --- | --- | --- | --- |
| **Age** (*M*, *SD*)^1^ | 22.41 (3.12) | 21.78 (3.0) | 24.00 (3.10) |
| **Height** (*M*, *SD*)^1^ | 172.20 (9.1) | 168.37 (6.90) | 181.89 (6.40) |
| **Education** (%, *n*)  Entrance qualification for universities of applied sciences  German university entrance qualification  Higher education qualification  Other | 9.38% (6)  75% (48)  14.06% (9)  1.56 (1) | 6.25% (4)  18.75% (12)  1.56% (1)  1.56% (1) | 3.13% (2)  56.25% (36)  12.50% (8)  0% (0) |
| **Weight** (*M*, *SD*)^1^ | 67.34 (13.92) | 62.67 (10.91) | 79.28 (13.89) |
| **BMI** (*M*, *SD*)^1^ | 22.62 (3.72) | 22.07 (3.39) | 23.98 (4.24) |
| **No Smoking** (%, *n*)^2^ | 74.60% (47)^1^ | 80.4 % (37)^7^ | 55.6 % (10) |
| **No hormonal birth control** (%, *n*)^2^ | 79.69 % (51) | 71.7 % (33) | / |
| **No Previous Drug Use** (%, *n*)^2^ | 69.84% (44)^1^ | 82.6 % (38)^7^ | 33.3% (6) |
| **NEO-FFI**^4^ (*M*, *SD*)^1^  Neuroticism  Extraversion  Openness  Conscientiousness  Agreeableness | 1.97 (.57)  2.56 (.62)  2.47 (.54)  2.76 (.75)  2.87 (.35) | 2.00 (.46)  2.61 (.59)  2.50 (.52)  2.85 (.74)  2.91 (.34) | 1.90 (.81)  2.44 (.72)  2.39 (.59)  2.55 (.73)  2.77 (.35) |
| **BSL-23**^5^ (*M*, *SD*)^1^ | 1.57 (.42) | 1.55 (.40) | 1.67 (.48) |
| **PANAS**^6^ (*M*, *SD*)^1^  Positive Affect  Negative Affect | 2.23 (.47)  2.50 (.49) | 2.24 (.44)  2.55 (.47) | 2.19 (.55)  2.37 (.53) |
| **SPF-IRI**^8^ (*M_Sum_*, *SD*)^3^  Fantasy  Empathic Concern  Perspective-Taking  Personal Distress | 12.92 (3.29)  13.33 (3.10)  14.25 (3.74)  10.09 (2.36) | 13.17 (3.29)  13.57 (3.14)  14.40 (3.81)  10.20 (2.36) | 12.28 (3.30)  12.72 (2.80)  13.89 (3.66)  9.83 (2.38) |
| **PID** *(M, SD)* ^2, 9, 10^  Negative Affect  Detachment  Antagonism  Disinhibition  Psychoticism | 2.64 (1.0)  2.52 (.98)  1.97 (.74)  2.48 (.86)  2.06 (.64) | 2.76 (.98)  2.47 (1.05)  1.90 (.77)  2.44 (.94)  1.98 (.66) | 2.40 (1.03)  2.62 (.85)  2.10 (.68)  2.56 (.70)  2.23 (.59) |

^1^ Body Mass Index, Mean, Standard Deviation; ^2^ Percentage, Number; ^3^ Mean Sum Score, Standard Deviation; ^4^ NEO-Five-Factor Inventory (Kanning, 2009); ^5^ Borderline-Symptom Checklist (BSL-23; Bohus et al., 2001); ^6^ Positive and Negative Affect Scale (PANAS-SF; Breyer & Bluemke, 2016; Watson et al., 1988); ^7^ Missing: 1.56 % (1), ^8^ Saarbrücker Persönlichkeitsfragebogen Empathie (Paulus, 2009); ^9^ The Personality Inventory for DSM-V (Brief Form, PID-V) (Krueger et al., 2012); ^10^ Missing: 17.5% (11)

**Table 2.** Correlations (Pearson’s *r*) of valence and arousal ratings per category (N = 64)

|  |  | **Correlation (Pearson’s *r*)** | | | | | | | | | | |
| --- | --- | --- | --- | --- | --- | --- | --- | --- | --- | --- | --- | --- |
|  |  | **Valence**^1^ | | | | |  | **Arousal**^1^ | | | | |
|  |  | NO |  | SIO |  | SSI |  | NO |  | SIO |  | SSI |
| **Valence** |  |  |  |  |  |  |  |  |  |  |  |  |
| NO |  | 1 |  | .50** |  | -.03 |  | .60** |  | .28* |  | .10 |
| SIO |  |  |  | 1 |  | .50** |  | .50** | - | .64** |  | .49** |
| SSI |  |  |  |  |  | 1 |  | .12 |  | .32** |  | .56** |
| **Arousal** |  |  |  |  |  |  |  |  |  |  |  |  |
| NO |  |  |  |  |  |  |  | 1 |  | .78** |  | .58** |
| SIO |  |  |  |  |  |  |  |  |  | 1 |  | .83** |
| SSI |  |  |  |  |  |  |  |  |  |  |  | 1 |

* Significant at *α* < .05, **significant at *α* < .01. ^1^ Valence and Arousal Ratings as measured by the Self-Assessment Manikin (SAM; Bradley & Lang, 1994).

**Laboratory Setting**

The mean temperature in the laboratory was *M* = 22.77°C (*n* = 57, *SD* = .81, Range: 21.00 to 23.00 °C), with a humidity of *M* = 39.92% (*SD* = 6.97, Range: 39.92, 59). Participants completed a short interview before the experiment, which included their current physiological state, caffeine/ alcohol intake, and stress anticipation. In the post-experimental interview, participants rated social desirability, concentration, physiological and psychological distress during the experiment, and boredom. Characteristics of the laboratory setting were rated after the experiment using a bipolar 4-point Likert Scale (feeling of insecurity in the laboratory 1 = not insecure, 4 = very insecure; brightness with 1 = very dark, 5 = very bright; air freshness with 1 = very sticky and 5 = very airy; temperature with 1 = very cold and 5 = very warm). Only 7.8% of participants rated feeling slightly insecure in the laboratory. Only four participants (6.3%) perceived the laboratory as very airy, and only two participants (3.2%) rated the temperature as very cold/ very warm. This indicates relatively stable and sufficiently good conditions of the laboratory environment across the participants.

**Table 3.** Laboratory Settings and Participants’ Ratings of the Laboratory

|  | **All (***N* = 64) | |
| --- | --- | --- |
|  | **Mean** | **SD** |
| Before the Experiment: |  |  |
| General bodily state^1^ | 2.17 | .66 |
| Reports of body pain^2^ | .30 | .82 |
| Sleep last night^1^ | 2.11 | .69 |
| Sleepiness^1^ | .21 | .60 |
| Coffeine/ Tea intake (yes) | 50% | / |
| Smoked before the experiment (yes) | 9.38% | / |
| Alcohol intake in the last 24 hours (yes) | 10.94% | / |
| Sports activity before the experiment (yes)t | 7.82 | / |
| Anticipation of picture aversiveness^1^ | 1.92 | .66 |
| After the experiment: |  |  |
| Social Desirability^1^ | 2.70 | .87 |
| Concentration Problems^1^ | 3.27 | .48 |
| Bodily Distress^1^ | 1.19 | .39 |
| Psychological Distress^1^ | 1.27 | .45 |
| Boredom^1^ | 1.86 | .56 |

^1^ Rated on a 4-point Likert Scale from 1 (not at all) to 4 (very); ^2^ Pain Rating from 0 to 10

**Emotional Picture Set of Self-Injury Images (EPSI) – Item Characteristics**

**Table 4.** Picture characteristics in terms of valence, arousal and z-standardized physiological variables (heart period, acoustic evoked startle response, skin conductance response

Category: 1 = Neutral (NO); 2 = Self-Injury Object (SIO), 3 = Scenes with Pre-Self-Injury (pre-SSI), 4 = Scenes with Post Self-Injury (post-SSI)

M = Mean, SD = Standard Deviation, ITC = Item-Total-Correlation

Valence: 9-point Likert Scale (0 = pleasant to 8 = unpleasant)

Arousal: 9-point Likert Scale (0 = calm to 8 = arousing)

HP_A: Heart Period, Acceleration (0-2s after Simulus Onset); z-standardized

HP_D: Heart Period, Deceleration (2-5s after Stimulus Onset), z-standardized

ASR: Acoustic Evoked Startle Reaction (max. Amplitude), z-standardized

SCR: Skin Conductance Response (max. Amplitude), z-standardized

| **Nr.** | **Category** | **Valence** | | | **Arousal** | | | **HP_A** | | **HP_D** | | **ASR** | | **SCR** | |
| --- | --- | --- | --- | --- | --- | --- | --- | --- | --- | --- | --- | --- | --- | --- | --- |
|  |  | **M** | **SD** | **ITC** | **M** | **SD** | **ITC** | **M** | **SD** | **M** | **SD** | **M** | **SD** | **M** | **SD** |
| 2 | 3 | 5.39 | 1.24 | .71 | 4.97 | 2.18 | .81 | 0.12 | 0.92 | 0.09 | 0.88 | -0.10 | 1.00 | 0.52 | 1.45 |
| 3 | 4 | 5.45 | 1.26 | .70 | 4.80 | 2.07 | .82 | -0.02 | 0.88 | 0.01 | 1.04 | -0.02 | 1.04 | 0.03 | 1.20 |
| 4 | 4 | 5.58 | 1.29 | .71 | 5.02 | 1.88 | .72 | 0.12 | 0.97 | 0.17 | 1.01 | 0.11 | 0.99 | 0.04 | 1.16 |
| 5 | 1 | 3.45 | 1.31 | .27 | 3.16 | 1.80 | .55 | -0.07 | 1.02 | -0.11 | 1.26 | 0.04 | 0.94 | -0.21 | 0.90 |
| 6 | 1 | 2.94 | 1.42 | .70 | 2.52 | 2.01 | .83 | 0.11 | 1.06 | -0.11 | 1.06 | 0.17 | 0.93 | -0.06 | 1.13 |
| 7 | 1 | 2.39 | 1.56 | .57 | 2.36 | 2.01 | .77 | -0.03 | 0.89 | 0.10 | 1.01 | -0.23 | 0.99 | -0.13 | 0.87 |
| 8 | 1 | 3.42 | 1.35 | .81 | 2.95 | 1.89 | .80 | -0.10 | 0.85 | 0.02 | 0.95 | -0.09 | 1.01 | -0.10 | 0.84 |
| 9 | 1 | 3.28 | 1.57 | .65 | 2.80 | 2.05 | .82 | 0.16 | 0.82 | 0.11 | 0.77 | 0.02 | 1.13 | -0.03 | 0.86 |
| 10 | 1 | 2.56 | 1.51 | .75 | 2.38 | 2.10 | .79 | -0.04 | 1.04 | -0.09 | 1.02 | -0.09 | 0.89 | 0.01 | 0.98 |
| 11 | 1 | 3.16 | 1.20 | .65 | 2.94 | 1.97 | .83 | -0.09 | 0.95 | -0.19 | 0.89 | -0.13 | 0.96 | -0.13 | 0.94 |
| 12 | 1 | 3.69 | 1.37 | .48 | 3.48 | 2.17 | .79 | -0.27 | 0.90 | -0.21 | 1.03 | 0.06 | 1.01 | 0.09 | 1.10 |
| 13 | 1 | 3.84 | 1.14 | .69 | 3.36 | 1.95 | .83 | 0.13 | 1.18 | 0.21 | 1.04 | 0.11 | 1.00 | -0.13 | 0.75 |
| 14 | 1 | 3.30 | 1.29 | .60 | 3.02 | 1.79 | .72 | -0.01 | 0.90 | -0.08 | 0.95 | -0.11 | 1.01 | -0.03 | 0.89 |
| 15 | 1 | 3.72 | 1.17 | .73 | 3.02 | 2.00 | .80 | -0.05 | 0.97 | -0.01 | 1.18 | -0.10 | 0.94 | -0.09 | 0.90 |
| 16 | 1 | 3.03 | 1.59 | .71 | 2.98 | 2.11 | .86 | 0.10 | 1.10 | 0.09 | 0.77 | 0.01 | 1.22 | -0.12 | 0.87 |
| 17 | 1 | 2.94 | 1.52 | .80 | 2.73 | 2.09 | .80 | 0.11 | 1.05 | 0.08 | 0.84 | 0.08 | 1.02 | -0.08 | 0.79 |
| 18 | 1 | 3.23 | 1.40 | .75 | 2.80 | 2.01 | .89 | 0.03 | 1.26 | 0.12 | 1.17 | 0.13 | 1.12 | 0.11 | 1.08 |
| 19 | 1 | 3.42 | 1.60 | .55 | 2.95 | 2.11 | .88 | 0.07 | 0.93 | -0.08 | 0.99 | 0.00 | 1.00 | -0.16 | 0.78 |
| 20 | 1 | 3.08 | 1.49 | .73 | 2.91 | 2.08 | .85 | -0.12 | 0.92 | -0.14 | 1.00 | -0.13 | 0.98 | -0.02 | 1.11 |
| 21 | 1 | 3.42 | 1.28 | .57 | 3.06 | 1.94 | .87 | -0.07 | 0.99 | 0.06 | 1.05 | 0.02 | 1.01 | -0.10 | 0.75 |
| 22 | 1 | 3.09 | 1.19 | .59 | 2.91 | 1.87 | .76 | -0.02 | 1.03 | -0.08 | 1.00 | -0.13 | 0.94 | -0.05 | 1.36 |
| 23 | 1 | 3.80 | 1.18 | .77 | 2.98 | 1.90 | .84 | -0.03 | 0.83 | -0.23 | 0.97 | -0.17 | 0.88 | -0.23 | 0.64 |
| 24 | 1 | 2.47 | 1.55 | .63 | 2.11 | 1.99 | .73 | -0.05 | 1.14 | -0.19 | 1.02 | -0.03 | 1.07 | -0.25 | 0.50 |
| 25 | 1 | 3.19 | 1.34 | .77 | 2.67 | 1.96 | .83 | 0.04 | 0.93 | 0.06 | 0.88 | -0.12 | 0.98 | 0.00 | 0.94 |
| 26 | 1 | 2.70 | 1.39 | .77 | 2.56 | 2.05 | .87 | -0.17 | 1.22 | 0.00 | 1.11 | 0.00 | 0.99 | -0.17 | 0.69 |
| 27 | 1 | 2.28 | 1.24 | .57 | 2.22 | 1.97 | .76 | -0.08 | 0.70 | -0.13 | 0.86 | 0.01 | 0.88 | -0.01 | 0.94 |
| 28 | 1 | 3.41 | 1.53 | .63 | 3.06 | 2.17 | .79 | 0.09 | 1.02 | 0.10 | 1.16 | 0.09 | 1.01 | -0.25 | 0.67 |
| **Nr.** | **Category** | **M** | **SD** | **ITC** | **M** | **SD** | **ITC** | **M** | **SD** | **M** | **SD** | **M** | **SD** | **M** | **SD** |
| 29 | 1 | 3.23 | 1.19 | .59 | 2.95 | 1.91 | .83 | 0.09 | 1.16 | -0.05 | 0.97 | 0.07 | 1.09 | -0.05 | 0.90 |
| 30 | 1 | 3.75 | 1.32 | .71 | 3.19 | 2.17 | .83 | -0.08 | 0.95 | -0.08 | 0.98 | 0.20 | 1.13 | -0.03 | 0.91 |
| 31 | 1 | 2.69 | 1.46 | .72 | 2.53 | 2.02 | .83 | -0.11 | 1.12 | 0.07 | 0.90 | 0.02 | 1.05 | -0.06 | 1.00 |
| 32 | 1 | 3.56 | 1.04 | .48 | 3.17 | 1.92 | .78 | -0.04 | 1.00 | -0.08 | 0.86 | 0.18 | 0.99 | -0.06 | 0.98 |
| 33 | 1 | 3.45 | 1.19 | .55 | 3.06 | 1.89 | .87 | 0.14 | 0.99 | 0.31 | 0.97 | -0.12 | 1.08 | -0.01 | 0.97 |
| 34 | 1 | 2.89 | 1.50 | .54 | 2.58 | 1.90 | .74 | 0.03 | 0.82 | 0.05 | 0.98 | 0.04 | 1.04 | -0.20 | 0.72 |
| 35 | 1 | 3.75 | 1.17 | .69 | 3.41 | 2.02 | .78 | -0.01 | 0.92 | -0.14 | 0.90 | 0.11 | 1.11 | -0.13 | 0.74 |
| 36 | 1 | 2.97 | 1.64 | .77 | 2.83 | 2.12 | .89 | -0.04 | 1.02 | 0.02 | 0.92 | 0.02 | 0.98 | -0.07 | 0.80 |
| 37 | 1 | 2.50 | 1.54 | .71 | 2.45 | 2.08 | .78 | -0.01 | 1.05 | -0.02 | 0.97 | -0.08 | 1.02 | -0.17 | 0.77 |
| 38 | 1 | 3.56 | 1.10 | .59 | 3.20 | 1.98 | .80 | -0.11 | 0.99 | -0.06 | 1.10 | 0.01 | 0.99 | -0.07 | 0.87 |
| 39 | 1 | 3.42 | 1.35 | .54 | 3.02 | 1.93 | .67 | 0.20 | 1.11 | 0.08 | 1.07 | -0.18 | 0.85 | -0.08 | 0.77 |
| 40 | 1 | 3.66 | 1.25 | .72 | 3.17 | 2.14 | .86 | 0.05 | 1.09 | -0.03 | 0.98 | 0.21 | 0.94 | -0.15 | 0.70 |
| 41 | 1 | 3.64 | 1.25 | .59 | 3.45 | 1.94 | .84 | 0.02 | 0.88 | 0.17 | 0.96 | 0.19 | 1.04 | -0.20 | 0.69 |
| 42 | 1 | 3.31 | 1.07 | .59 | 2.83 | 1.92 | .84 | 0.27 | 1.00 | 0.17 | 1.04 | 0.23 | 1.10 | -0.01 | 1.03 |
| 43 | 1 | 3.09 | 1.15 | .59 | 2.44 | 1.86 | .82 | -0.10 | 1.00 | -0.10 | 1.02 | 0.05 | 0.92 | -0.25 | 0.58 |
| 44 | 1 | 2.67 | 1.32 | .62 | 2.38 | 1.89 | .75 | 0.02 | 0.98 | -0.12 | 0.80 | 0.08 | 1.02 | -0.13 | 1.32 |
| 45 | 1 | 3.23 | 1.35 | .73 | 2.64 | 2.01 | .82 | -0.12 | 0.73 | -0.12 | 0.68 | 0.18 | 1.02 | 0.02 | 1.13 |
| 46 | 1 | 2.88 | 1.30 | .70 | 2.50 | 1.79 | .69 | -0.24 | 0.78 | -0.09 | 0.79 | -0.08 | 1.03 | -0.15 | 0.82 |
| 47 | 1 | 2.59 | 1.54 | .58 | 2.91 | 2.14 | .67 | 0.00 | 1.05 | 0.02 | 1.06 | 0.19 | 0.92 | -0.04 | 0.92 |
| 48 | 1 | 3.47 | 1.25 | .74 | 3.11 | 1.97 | .87 | -0.31 | 0.98 | -0.13 | 0.91 | -0.13 | 0.89 | -0.16 | 0.67 |
| 49 | 1 | 2.22 | 1.52 | .55 | 2.50 | 2.01 | .74 | -0.21 | 0.88 | -0.22 | 1.01 | 0.21 | 1.03 | -0.04 | 0.72 |
| 50 | 2 | 4.14 | 1.15 | .42 | 3.80 | 2.12 | .83 | 0.03 | 1.09 | -0.02 | 1.00 | 0.29 | 0.97 | 0.23 | 1.27 |
| 51 | 2 | 4.66 | 1.09 | .65 | 4.16 | 1.94 | .82 | -0.07 | 0.82 | -0.17 | 0.87 | -0.07 | 0.92 | 0.12 | 1.12 |
| 52 | 2 | 4.69 | 1.32 | .72 | 4.38 | 2.12 | .83 | 0.04 | 1.00 | 0.04 | 0.99 | 0.08 | 0.92 | -0.09 | 0.75 |
| 53 | 2 | 4.81 | 1.18 | .64 | 4.42 | 2.02 | .85 | 0.08 | 1.24 | 0.03 | 0.94 | -0.04 | 0.95 | -0.11 | 0.86 |
| 54 | 2 | 4.75 | 1.18 | .60 | 4.25 | 2.00 | .89 | 0.00 | 0.99 | 0.03 | 1.06 | 0.15 | 1.11 | 0.00 | 1.05 |
| 55 | 2 | 4.44 | 1.18 | .54 | 3.83 | 2.00 | .79 | 0.03 | 0.99 | -0.06 | 0.91 | -0.04 | 0.93 | -0.02 | 0.86 |
| 56 | 2 | 4.44 | 1.01 | .71 | 4.16 | 1.98 | .91 | 0.01 | 0.98 | 0.03 | 0.95 | -0.12 | 0.97 | 0.21 | 1.31 |
| 57 | 2 | 3.86 | 1.15 | .63 | 3.34 | 1.97 | .77 | -0.22 | 1.06 | -0.19 | 1.02 | -0.08 | 1.06 | -0.21 | 0.64 |
| 58 | 2 | 4.81 | 1.58 | .48 | 4.05 | 1.90 | .77 | -0.04 | 1.05 | 0.03 | 0.99 | -0.14 | 0.90 | -0.09 | 0.67 |
| 59 | 2 | 4.34 | 1.42 | .53 | 4.05 | 2.04 | .80 | -0.09 | 0.98 | 0.05 | 0.96 | 0.02 | 0.98 | -0.19 | 0.65 |
| 60 | 2 | 4.06 | 0.92 | .63 | 3.77 | 1.93 | .81 | 0.00 | 0.85 | -0.04 | 0.98 | -0.02 | 0.98 | -0.15 | 0.68 |
| 61 | 2 | 4.41 | 1.31 | .54 | 4.28 | 2.06 | .89 | 0.12 | 1.07 | 0.11 | 1.09 | -0.09 | 0.99 | 0.09 | 1.27 |
| 62 | 2 | 4.41 | 1.39 | .67 | 4.17 | 2.14 | .85 | 0.02 | 0.90 | -0.01 | 0.94 | 0.00 | 1.00 | 0.03 | 1.04 |
| 63 | 4 | 5.61 | 1.36 | .69 | 4.95 | 2.10 | .82 | 0.15 | 1.02 | 0.06 | 0.92 | -0.23 | 1.02 | 0.11 | 1.10 |
| 64 | 4 | 5.86 | 1.21 | .89 | 5.06 | 2.10 | .93 | 0.05 | 1.00 | -0.04 | 0.93 | -0.07 | 0.89 | 0.02 | 0.91 |
| 65 | 4 | 5.80 | 1.22 | .84 | 4.94 | 2.25 | .93 | -0.01 | 1.03 | 0.05 | 1.03 | -0.08 | 0.96 | 0.01 | 0.85 |
| 66 | 4 | 5.89 | 1.13 | .71 | 5.25 | 2.04 | .78 | 0.05 | 0.90 | 0.02 | 0.91 | -0.31 | 0.94 | 0.22 | 1.21 |
| 67 | 4 | 5.89 | 1.22 | .83 | 5.27 | 2.34 | .93 | 0.00 | 0.92 | -0.17 | 1.11 | 0.02 | 1.07 | 0.32 | 1.28 |
| 68 | 4 | 5.83 | 1.28 | .84 | 5.20 | 1.95 | .84 | -0.07 | 1.05 | -0.02 | 1.26 | -0.13 | 0.88 | 0.10 | 1.01 |
| 69 | 4 | 6.08 | 1.41 | .70 | 5.61 | 2.08 | .85 | -0.03 | 0.91 | -0.08 | 1.00 | 0.07 | 1.08 | 0.71 | 1.60 |
| 70 | 4 | 5.56 | 1.31 | .74 | 4.89 | 2.01 | .86 | 0.17 | 1.05 | 0.18 | 0.89 | -0.01 | 0.87 | 0.08 | 0.89 |
| 71 | 3 | 5.28 | 1.17 | .76 | 4.73 | 1.94 | .76 | -0.03 | 0.79 | 0.23 | 1.10 | 0.10 | 0.98 | 0.13 | 1.17 |
| 72 | 3 | 5.27 | 1.06 | .76 | 4.64 | 2.00 | .90 | 0.21 | 1.00 | 0.29 | 1.06 | 0.14 | 0.97 | 0.33 | 1.34 |
| 73 | 3 | 5.20 | 1.26 | .71 | 4.66 | 1.84 | .88 | 0.06 | 0.98 | -0.03 | 0.86 | 0.03 | 0.91 | 0.00 | 0.90 |
| **Nr.** | **Category** | **M** | **SD** | **ITC** | **M** | **SD** | **ITC** | **M** | **SD** | **M** | **SD** | **M** | **SD** | **M** | **SD** |
| 74 | 3 | 5.19 | 1.08 | .72 | 4.61 | 2.13 | .90 | -0.03 | 1.06 | 0.10 | 0.99 | 0.02 | 1.06 | 0.06 | 1.42 |
| 75 | 3 | 5.44 | 1.11 | .78 | 4.89 | 1.97 | .90 | 0.06 | 1.01 | -0.08 | 0.83 | -0.11 | 0.94 | 0.03 | 0.89 |
| 76 | 4 | 5.50 | 1.22 | .70 | 5.13 | 1.86 | .72 | -0.07 | 1.33 | 0.12 | 1.20 | 0.25 | 1.20 | 0.03 | 0.82 |
| 77 | 4 | 5.72 | 1.17 | .74 | 5.05 | 2.01 | .90 | -0.06 | 1.02 | -0.05 | 0.90 | -0.01 | 1.03 | 0.15 | 1.16 |
| 78 | 4 | 5.59 | 1.24 | .82 | 4.97 | 2.11 | .88 | -0.05 | 0.94 | 0.01 | 1.03 | 0.04 | 1.03 | 0.00 | 0.91 |
| 79 | 4 | 5.70 | 1.16 | .71 | 5.33 | 2.04 | .81 | -0.05 | 0.84 | -0.04 | 0.94 | -0.12 | 0.91 | 0.28 | 1.16 |
| 80 | 4 | 5.88 | 1.08 | .86 | 5.30 | 2.05 | .86 | 0.17 | 1.11 | 0.07 | 1.00 | -0.22 | 0.90 | -0.02 | 0.92 |
| 81 | 4 | 5.72 | 1.28 | .81 | 4.92 | 2.15 | .90 | -0.08 | 1.15 | -0.14 | 1.10 | 0.08 | 0.97 | 0.13 | 0.84 |
| 82 | 4 | 6.00 | 1.51 | .53 | 5.31 | 2.08 | .77 | -0.10 | 1.04 | 0.06 | 1.16 | 0.07 | 0.99 | 0.32 | 1.28 |
| 83 | 3 | 5.23 | 1.26 | .74 | 4.48 | 2.12 | .83 | -0.12 | 1.02 | -0.08 | 1.02 | -0.09 | 1.06 | -0.09 | 0.86 |
| 84 | 3 | 5.31 | 1.22 | .82 | 4.84 | 1.99 | .78 | -0.05 | 1.02 | -0.06 | 1.00 | -0.03 | 0.91 | 0.31 | 1.24 |
| 85 | 3 | 5.28 | 1.21 | .82 | 4.63 | 2.11 | .85 | 0.08 | 0.78 | 0.16 | 0.89 | -0.17 | 1.00 | -0.10 | 0.90 |
| 86 | 3 | 5.19 | 1.05 | .81 | 4.55 | 1.92 | .86 | -0.09 | 0.97 | -0.19 | 1.05 | -0.17 | 0.94 | 0.01 | 1.10 |
| 87 | 3 | 5.44 | 1.22 | .81 | 4.97 | 2.06 | .82 | 0.05 | 0.99 | -0.02 | 1.07 | 0.01 | 0.95 | 0.17 | 0.92 |
| 88 | 3 | 5.22 | 1.33 | .65 | 4.63 | 1.99 | .84 | 0.31 | 1.06 | 0.44 | 0.95 | -0.01 | 0.98 | 0.14 | 1.21 |
| 89 | 4 | 5.45 | 1.28 | .77 | 4.81 | 2.24 | .71 | 0.21 | 1.08 | 0.24 | 1.29 | 0.19 | 0.96 | 0.32 | 1.34 |
| 90 | 3 | 5.13 | 1.15 | .63 | 4.66 | 1.88 | .84 | 0.07 | 1.00 | -0.10 | 1.00 | 0.02 | 1.02 | -0.01 | 1.12 |

**Table 5.** Multilevel Models of Valence (with significances marked with *)

| **Model** | ***b*-Estimate**^3^ | **Std. Err.**^3^ | **df**^4^ | ***t*-value** | ***p*-value**^5^ | **CI**^6^ | **ICC**^7^ | **AIC/ BIC**^8^ | **Contrasts**^9^ | **Power**  **[95% CI]** |
| --- | --- | --- | --- | --- | --- | --- | --- | --- | --- | --- |
| **Unconditional Random Intercept Model (Null Model): *Valence ~ 1+(1\|ID*^1^*)*** | | | | | | | | | | |
| ID^1^ | 4.17 | .07 | 63 | 53.97 | <.001*** | [4.03, 4.33] | .12 | AIC: 21813  BIC: 21833 | / | / |
| **Conditional Random Intercept Model (M1): *Valence ~ Category +Trial +Startle + (1\|ID)*** | | | | | | | | | | |
| Intercept  SIO^2^  SSI^2^  Trial  Startle | 3.13  1.28  2.38  .00  .08 | .09  .05  .03  .00  .03 | 92.48  5628.00  5628.00  5628.00  5628.00 | 36.74  27.31  68.14  -.30  2.598 | <.001***  <.001***  <.001***  .76  .01** | [2.96, 3.30]  [1.19, 1.38]  [2.31, 2.44]  [.00, .00]  [.02, .14] | .21 | AIC: 18404  BIC: 18451 | *t* _NO-SIO_ (5628) = -27.31, *p* < .001***  *t* _NO-SSI_ (5628) = -68.14, *p* <.001***  *t* _SIO-SSI_ (5628) = -22.12, *p* <.001*** | 100%  [96.38, 100] |
| **Conditional Random Intercept Model with Interactions (M2): *Valence ~ Category + Trial + Startle + Trial:Category + Startle:Category + (1\|ID)*** | | | | | | | | | | |
| Intercept  SIO^2^  SSI^2^  Trial  Startle  Interaction SIO-Trial  Interaction SSI-Trial  Interaction SIO-Startle  Interaction SSI-Startle | 3.04  1.55  2.52  .00  .13  .00  .00  -.15  -.07 | .09  .11  .08  .00  .04  .00  .00  .09  .07 | 118.85  5628.77  5628.55  5626.21  5624.12  5631.84  5628.11  5624.50  5624.05 | 33.52  14.40  32.09  1.48  2.89  -2.32  -1.81  -1.62  -1.06 | <.001***  <.001***  <.001***  .14  <.001***  .02*  .07  .11  .29 | [2.86, 3.22]  [1.34, 1.77]  [2.37, 2.68]  [.00, .00]  [.04, .22]  [-.01, -.00]  [-.01, -.00]  [-.34, -.03]  [-.21, .06] | .21 | AIC: 18403  BIC: 18476 | *t* _NO-SIO_ (5624) = -27.35, *p* < .001***  *t* _NO-SSI_ (5624) = -68.14, *p* <.001***  *t* _SIO-SSI_ (5624) = -22.10, *p* <.001*** | 100%  [96.38, 100] |
| **Conditional Random Intercept Random Slope Model (M3): *Valence ~ Category + Trial + Startle + Trial:Category + Startle:Category + (1+Category\|ID)*** | | | | | | | | | | |
| Intercept  SIO^2^  SSI^2^  Trial  Startle  Interaction SIO-Trial  Interaction SSI-Trial  Interaction SIO-Startle  Interaction SSI-Startle | 3.03  1.57  2.54  .00  .13  .00  .00  -.16  -.07 | .12  .13  .17  .00  .04  .00  .00  .08  .06 | 79.86  190.83  82.90  5502.82  5498.32  5448.92  5508.26  5510.60  5498.17 | 27.34  11.98  14.79  2.05  3.33  -2.79  -2.36  -1.96  -1.22 | <.001***  <.001***  <.001***  .04*  <.001***  .01*  02*  .05  .23 | [2.80, 3.26]  [1.32, 1.83]  [2.20, 2.87]  [.00, .00]  [.05, .20]  [-.01, -.00]  [-.01, -.00]  [-.32, .00]  [-.19, .04] | .40 | AIC: 17110  BIC: 17217 | *t* _NO-SIO_ (63) = -13.02, *p* < .001***  *t* _NO-SSI_ (63) = -14.85, *p* <.001***  *t* _SIO-SSI_ (63) = -9.74, *p* <.001*** | 100%  [96.38, 100] |
| **Conditional Random Intercept Model with Factor Self-Injury (M4): *Valence ~ Category + Trial + Startle + Post-Self-Injury + Trial:Category + Startle:Category + (1+Category\|ID*** | | | | | | | | | | |
| Intercept  SIO^2^  SSI^2^  Trial  Startle  Post-Self-Injury  Interaction SIO-Trial  Interaction SSI-Trial  Interaction SIO-Startle  Interaction SSI-Startle | 3.03  1.57  2.28  .00  .13  .46  .00  .00  -.16  -.07 | .12  .13  .17  .00  .04  .05  .00  .00  .08  .06 | 79.56  188.26  86.46  5501.73  5497.31  5497.07  5454.17  5507.09  5509.39  5497.16 | 26.08  12.05  13.18  2.06  3.34  9.74  -2.82  -2.55  -1.97  -1.21 | <.001***  <.001***  <.001***  .04*  <.001***  <.001***  <.001***  .01*  .05  .23 | [2.80, 3.26]  [1.32, 1.83]  [1.94, 2.62]  [.00, .00]  [.05, .20]  [.36, .55]  [-.01, -.00]  [-.01, -.00]  [-.32, .00]  [-.19, .04] | .41 | AIC: 17018  BIC: 17131 | *t* _NO-SIO_ (63) = -13.02, *p* < .001***  *t* _NO-SSI_ (66.7) = -13.01, *p* <.001***  *t* _SIO-SSI_ (70.7) = -7.17, *p* <.001*** | 100%  [96.38, 100] |

^1^ ID: Subjects; ^2^ Category: Self-Injury Objects, Scenes with Self-Injury;^,3^ *b*-Estimates and standard errors; ^4^ Satterthwaite; ^5^ two-tailed *p*-value; ^6^ 95% bootstrapped CI; ^7^ Adjusted Intraclass Correlation Coefficient; ^8^ Model Fit Indices,^9^ Direct Group Comparisons

**Table 6.** Multilevel Models of Arousal (with significances marked with *)

| **Model** | ***b*-Estimate**^3^ | **Std. Err.**^3^ | **Df**^4^ | ***t*-value** | ***p*-value**^5^ | **CI**^6^ | **ICC**^7^ | **AIC/ BIC**^8^ | **Contrasts**^9^ | **Power**  **[95% CI]** |
| --- | --- | --- | --- | --- | --- | --- | --- | --- | --- | --- |
| **Unconditional Random Intercept Model (Null Model): *Arousal ~ 1+(1\|ID*^1^*)*** | | | | | | | | | | |
| ID^1^ | 3.75 | .19 | 63 | 20.02 | <.001*** | [3.38, 4.12] | .44 | AIC: 2368  BIC: 22388 | / | / |
| **Conditional Random Intercept Model (M1): *Arousal ~ Category +Trial +Startle + (1\|ID)*** | | | | | | | | | | |
| Intercept  SIO^2^  SSI^2^  Trial  Startle | 2.79  1.20  2.09  .00  .24 | .19  .05  .04  .00  .04 | 69.18  5628  5628  5628  5628 | 14.51  22.21  52.12  -1.79  6.65 | <.001***  <.001***  <.001***  .07  <.001*** | [2.41, 3.16]  [1.10, 1.31]  [2.01, 2.17]  [-.00, .00]  [.17, .31] | .54 | AIC: 20102  BIC: 20149 | *t* _NO-SIO_ (5628) = -22.21, *p* < .001***  *t* _NO-SSI_ (5628) = -52.12, *p* <.001***  *t* _SIO-SSI_ (5628) = -15.66, *p* <.001*** | 100%  [96.38, 100] |
| **Conditional Random Intercept Model with Interactions (M2): *Arousal ~ Category + Trial + Startle + Trial:Category + Startle:Category + (1\|ID)*** | | | | | | | | | | |
| Intercept  SIO^2^  SSI^2^  Trial  Startle  Interaction SIO-Trial  Interaction SSI-Trial  Interaction SIO-Startle  Interaction SSI-Startle | 2.67  1.46  2.33  .00  .35  .00  .00  -.19  -.23 | .20  .12  .09  .00  .05  .00  .00  .11  .08 | 75.11  5625.09  5624.58  5624.50  5624.03  5625.82  5624.94  5624.11  5624.01 | 13.66  11.73  25.76  .20  6.81  -1.67  -1.79  -1.73  -2.85 | <.001***  <.001***  <.001***  .84  <.001***  .09  .07  .08  . <.001*** | [2.28, 3.05]  [1.21, 1.70]  [2.15, 2.51]  [-.00, .00]  [.25, .45]  [-.01, .00]  [-.01, .00]  [-.40, .03]  [-.39, -.07] | .54 | AIC: 20097  BIC: 20170 | *t* _NO-SIO_ (5624) = -22.24, *p* < .001***  *t* _NO-SSI_ (5624) = -52.10, *p* <.001***  *t* _SIO-SSI_ (5624) = -15.60, *p* <.001*** | 100%  [96.38, 100] |
| **Conditional Random Intercept Random Slope Model (M3): *Arousal ~ Category + Trial + Startle + Trial:Category + Startle:Category + (1+Category\|ID)*** | | | | | | | | | | |
| Intercept  SIO^2^  SSI^2^  Trial  Startle  Interaction SIO-Trial  Interaction SSI-Trial  Interaction SIO-Startle  Interaction SSI-Startle | 2.66  1.46  2.35  .00  .34  .00  .00  -.21  -.22 | .20  .16  .19  .00  .04  .00  .00  .10  .07 | 69.65  173.29  85.06  5502.08  5498.25  5497.46  5507.29  5508.72  5498.12 | 13.01  9.28  12.31  .52  7.68  -1.81  -2.37  -2.20  -3.19 | <.001***  <.001***  <.001***  .60  <.001***  .07  .02*  .03*  <.001*** | [2.53, 3.06]  [1.15, 1.77]  [1.97, 2.72]  [-.00, .00]  [.26, .43]  [-.01, .00]  [-.00, -.00]  [-.40, -.02]  [-.36, -.09] | .65 | AIC: 18908  BIC: 19015 | *t* _NO-SIO_ (63) = -9.86, *p* < .001***  *t* _NO-SSI_ (63) = -11.80, *p* <.001***  *t* _SIO-SSI_ (63) = -7.34, *p* <.001*** | 100%  [96.38, 100] |
| **Conditional Random Intercept Model with Factor Self-Injury (M4): *Arousal ~ Category + Trial + Startle + Post-Self-Injury + Trial:Category + Startle:Category + (1+Category\|ID)*** | | | | | | | | | | |
| Intercept  SIO^2^  SSI^2^  Trial  Startle  Post-Self-Injury  Interaction SIO-Trial  Interaction SSI-Trial  Interaction SIO-Startle  Interaction SSI-Startle | 2.66  1.46  2.13  .00  .34  .39  .00  .00  -.21  -.22 | .20  .16  .19  .00  .04  .05  .00  .00  .09  .07 | 69.59  172.11  89.24  5501.05  5497.25  5497.04  5498.72  5506.21  5507.62  5497.12 | 13.01  9.29  11.03  .53  7.71  7.18  -1.82  -2.51  -2.21  -3.21 | <.001***  <.001***  <.001***  .60  <.001***  <.001***  .07  .01*  .03*  <.001*** | [2.25, 3.06]  [1.15, 1.77]  [1.75, 2.51]  [-.00, .00]  [.26, .43]  [.29, .50]  [-.01, -.00]  [-.01, -.00]  [-.40, -.02]  [-.36, -.09] | .65 | AIC: 18859  BIC: 18972 | *t* _NO-SIO_ (63) = -9.86, *p* < .001***  *t* _NO-SSI_ (67.1) = -10.35, *p* <.001***  *t* _SIO-SSI_ (72) = -5.27, *p* <.001*** | 100%  [96.38, 100] |

^1^ ID: Subjects; ^2^ Category: Self-Injury Objects, Scenes with Self-Injury;^,3^ *b*-Estimates and standard errors; ^4^ Satterthwaite; ^5^ two-tailed *p*-value; ^6^ 95% bootstrapped CI; ^7^ Adjusted Intraclass Correlation Coefficient; ^8^ Model Fit Indices,^9^ Direct Group Comparisons

**Table 7.** Multilevel Models of Heart Period, Component A (with significances marked with *)

| **Model** | ***b*-Estimate**^3^ | **Std. Err.** ^3^ | **df**^4^ | ***t*-value** | ***p*-value**^5^ | **CI**^6^ | **ICC**^7^ | **AIC/ BIC**^8^ | **Contrasts**^9^ | **Power**  **[95% CI]** |
| --- | --- | --- | --- | --- | --- | --- | --- | --- | --- | --- |
| **Unconditional Random Intercept Model (Null Model): *HP_A ~ 1+(1\|ID*^1^*)*** | | | | | | | | | | |
| ID^1^ | 19.64 | 2.96 | 62.97 | 6.65 | <.001*** | [13.80, 25.47] | .05 | AIC: 67963  BIC: 67983 | / | / |
| **Conditional Random Intercept Model (M1): *HP_A ~ Category + Trial + Startle + (1\|ID)*** | | | | | | | | | | |
| Intercept  SIO^2^  SSI^2^  Trial  Startle | 29.52  2.66  4.47  -.32  4.86 | 4.07  3.70  2.74  .05  2.49 | 224.98  5618.98  5619.03  5619.01  5619.01 | 7.25  .72  1.63  -6.52  1.95 | <.001***  .47  .10  <.001***  .05 | [21.54, 37.51]  [-4.59, 9.90]  [-.91, 9.85]  [-.41, -.22]  [.02, 9.75] | .05 | AIC: 67922  BIC: 67968 | *t* _NO-SIO_ (5619) = -.72, *p* =.75  *t* _NO-SSI_ (5619) = -1.63, *p* =.23  *t* _SIO-SSI_ (5619) = -.47, *p* =.89 | 25%  [16.88, 34.66] |
| **Conditional Random Intercept Model with Interactions (M2): *HP_A ~ Category + Trial + Startle + Trial:Category + Startle:Category + (1\|ID)*** | | | | | | | | | | |
| Intercept  SIO^2^  SSI^2^  Trial  Startle  Interaction SIO-Trial  Interaction SSI-Trial  Interaction SIO-Startle  Interaction SSI-Startle | 28.94  .04  7.35  -.27  1.95  -.03  -.12  8.34  4.84 | 4.76  8.48  6.19  .07  3.50  .14  .11  7.40  5.49 | 407.36  5633.82  5625.44  5623.93  5615.49  5644.52  5631.29  5617.07  5615.28 | 6.08  .00  1.19  -3.99  .56  -.24  -1.09  1.13  .88 | <.001***  1.00  .24  <.001***  .58  .81  .28  .26  .38 | [19.62, 38.25]  [-16.60, 16.65]  [-4.78, 19.45]  [-.40, -.14]  [-4.91, 8.81]  [-.32, .25]  [-.33, .09]  [-6.15, 22.84]  [-5.91, 15.50] | .06 | AIC: 67927  BIC: 68000 | *t* _NO-SIO_ (5615) = -.72, *p* =.75  *t* _NO-SSI_ (5615) = -1.64, *p* =.23  *t* _SIO-SSI_ (5615) = -.47, *p* =.89 | 11%  [5.62, 18.83] |
| **Conditional Random Intercept Random Slope Model (M3): *HP_A ~ Category + Trial + Startle + Trial:Category + Startle:Category + (1+Category\|ID)*** | | | | | | | | | | |
| Intercept  SIO^2^  SSI^2^  Trial  Startle  Interaction SIO-Trial  Interaction SSI-Trial  Interaction SIO-Startle  Interaction SSI-Startle | 29.07  -.07  7.58  -.27  1.95  -.03  -.12  8.33  4.84 | 4.53  8.51  6.52  .07  3.49  .14  .11  7.38  5.47 | 379.40  3298.33  746.06  5590.01  5554.84  5382.80  5592.16  5554.53  5553.35 | 6.41  -.01  1.16  -4.04  .56  -.22  -1.14  1.13  .89 | <.001***  .99  .24  <.001***  .58  .83  .25  .26  .38 | [20.19, 37.94]  [-16.73, 16.61]  [-5.17, 20.34]  [-.41, -.14]  [-4.89, 8.78]  [-.31, .25]  [-.33, .09]  [-6.12, 22.78]  [-5.87, 15.56] | .06  Singularity | AIC:67923  BIC: 68029 | *t* _NO-SIO_ (62.9) = -.70, *p* =.76  *t* _NO-SSI_ (63) = -1.31, *p* =.40  *t* _SIO-SSI_ (63) = -.45, *p* =.89 | 21%  [13.49, 30.29] |
| **Conditional Random Intercept Model with Factor Self-Injury (M4): *HP_A ~ Category + Trial + Startle + Post-Self-Injury + Trial:Category + Startle:Category + (1\|ID)*** | | | | | | | | | | |
| Intercept  SIO^2^  SSI^2^  Trial  Startle  Post-Self-Injury  Interaction SIO-Trial  Interaction SSI-Trial  Interaction SIO-Startle  Interaction SSI-Startle | 28.94  .04  8.62  -.27  1.95  -2.28  -.03  -.12  8.34  4.84 | 4.76  8.48  6.63  .07  3.50  4.28  .14  .11  7.40  5.49 | 407.47  5632.82  5622.97  5622.93  5614.49  5614.00  5643.52  5630.30  5616.07  5614.28 | 6.08  .00  1.30  -3.99  .56  -.53  -.24  -1.08  1.13  .88 | <.001***  1.00  .19  <.001***  .58  .59  .81  .28  .26  .38 | [19.62, 38.25]  [-16.58, 16.65]  [-4.78, 19.47]  [-.40, -.14]  [-4.91, 8.81]  [-.32, .25]  [-.33, .25]  [-.33, .09]  [-6.15, 22.84]  [-5.91, 15.59] | .05 | AIC: 67929  BIC: 68008 | *t* _NO-SIO_ (5614) = -.72, *p* =.75  *t* _NO-SSI_ (5614) = -1.57, *p* =.26  *t* _SIO-SSI_ (5614) = -.68, *p* =.78 | 24%  [16.02, 33.57] |

^1^ ID: Subjects; ^2^ Category: Self-Injury Objects, Scenes with Self-Injury;^,3^ *b*-Estimates and standard errors; ^4^ Satterthwaite; ^5^ two-tailed *p*-value; ^6^ 95% bootstrapped CI; ^7^ Adjusted Intraclass Correlation Coefficient; ^8^ Model Fit Indices,^9^ Direct Group Comparisons

**Table 8.** Multilevel Models of Heart Period, Component D (with significances marked with *)

| **Model** | ***b*-Estimate**^3^ | **Std. Err.** ^3^ | **df**^4^ | ***t*-value** | ***p*-value**^5^ | **CI**^6^ | **ICC**^7^ | **AIC/ BIC**^8^ | **Contrasts**^9^ | **Power**  **[95% CI]** |
| --- | --- | --- | --- | --- | --- | --- | --- | --- | --- | --- |
| **Unconditional Random Intercept Model (Null Model): *HP_D ~ 1+(1\|ID*^1^*)*** | | | | | | | | | | |
| ID^1^ | 10.81 | 2.16 | 62.99 | 5.01 | <.001*** | [6.55, 15.08] | .05 | AIC: 64251  BIC: 64271 | / | / |
| **Conditional Random Intercept Model (M1): *HP_D ~ Category + Trial + Startle + (1\|ID)*** | | | | | | | | | | |
| Intercept  SIO^2^  SSI^2^  Trial  Startle | 20.66  1.04  4.82  -.28  2.15 | 2.96  2.66  1.97  .03  1.79 | 219.04  5619.00  5619.04  5619.03  5619.03 | 6.99  .39  2.44  -8.10  1.20 | <.001***  .70  .01*  <.001***  .23 | [14.86, 26.45]  [-4.17, 6.26]  [.95, 8.69]  [-.35, -.21]  [-1.36, 5.67] | .05 | AIC: 64186  BIC: 64232 | *t* _NO-SIO_ (5619) = -.39, *p* =.92  *t* _NO-SSI_ (5619) = -2.44, *p* =.04*  *t* _SIO-SSI_ (5619) = -1.35, *p* =.37 | 58%  [47.71, 67.80] |
| **Conditional Random Intercept Model with Interactions (M2): *HP_D ~ Category + Trial + Startle + Trial:Category + Startle:Category + (1\|ID)*** | | | | | | | | | | |
| Intercept  SIO^2^  SSI^2^  Trial  Startle  Interaction SIO-Trial  Interaction SSI-Trial  Interaction SIO-Startle  Interaction SSI-Startle | 16.51  4.22  15.66  -.17  .76  -.16  -.25  8.26  .41 | 3.44  6.10  4.45  .05  2.52  .10  .08  5.32  3.95 | 394.11  5633.35  5625.19  5623.72  5615.51  5643.83  5630.89  5617.05  5615.31 | 4.80  .69  3.52  -3.58  .30  -1.55  -3.18  1.55  .10 | <.001***  .49  <.001***  <.001***  .76  .12  <.001***  .12  .92 | [9.77, 23.24]  [-7.73, 16.17]  [6.93, 24.37]  [-.27, -.08]  [-4.17, 5.69]  [-.37, .04  [-.40, -.09]  [-2.16, 18.69]  [-7.31, 8.14] | .05 | AIC: 64181  BIC: 64254 | *t* _NO-SIO_ (5615) = -.41, *p* =.91  *t* _NO-SSI_ (5615) = -2.42, *p* =.04*  *t* _SIO-SSI_ (5615) = -1.32, *p* =.39 | 87%  [78.80, 92.89] |
| **Conditional Random Intercept Random Slope Model (M3): *HP_D ~ Category + Trial + Startle + Trial:Category + Startle:Category + (1+Category\|ID)*** | | | | | | | | | | |
| Intercept  SIO^2^  SSI^2^  Trial  Startle  Interaction SIO-Trial  Interaction SSI-Trial  Interaction SIO-Startle  Interaction SSI-Startle | 16.55  4.43  15.61  -.18  .74  -.17  -.25  8.36  .42 | 3.34  6.16  4.76  .05  2.51  .10  .08  5.30  3.93 | 330.34  1772.54  645.32  5591.62  5554.86  5438.28  5587.20  5552.91  5553.36 | 4.96  .72  3.28  -3.61  .30  -1.61  -3.18  1.58  .11 | <.001***  .47  <.001***  <.001***  .77  .11  <.001***  .11  .91 | [10.03, 23.07]  [-7.63, 16.48]  [6.28, 24.94]  [-.27, -.08]  [-4.17, 5.65]  [-.37, -.04]  [-40, -.09]  [-2.00, 18.73]  [-7.28, 8.12] | Singularity | AIC: 64169  BIC: 64275 | *t* _NO-SIO_ (62.9) = -.38, *p* =.92  *t* _NO-SSI_ (63) = -1.82, *p* =.17  *t* _SIO-SSI_ (63) = -1.22, *p* =.44 | 77%  [67.51, 84.83] |
| **Conditional Random Intercept Model with Factor Self-Injury (M4): *HP_D ~ Category + Trial + Startle + Post-Self-Injury + Trial:Category + Startle:Category + (1\|ID)*** | | | | | | | | | | |
| Intercept  SIO^2^  SSI^2^  Trial  Startle  Post-Self-Injury  Interaction SIO-Trial  Interaction SSI-Trial  Interaction SIO-Startle  Interaction SSI-Startle | 16.51  4.22  16.19  -.17  .76  -.96  -.16  -.25  8.26  .41 | 3.44  6.10  4.77  .02  2.52  3.08  .10  .08  5.32  3.95 | 394.19  5632.35  5622.76  5622.72  5614.51  5614.04  5642.83  5629.90  5616.05  5614.31 | 4.80  .69  3.39  -3.58  .30  -.31  -1.55  -3.18  1.55  .10 | <.001***  .49  <.001***  <.001***  .76  .75  .12  <.001***  .12  .92 | [9.77, 23.24]  [-7.73, 16.17]  [6.84, 25.54]  [-.27, -.08]  [-4.17, 5.69]  [-6.99, 5.97]  [-.37, .04]  [-.40, -.09]  [-2.16, 18.69]  [-7.32, 8.14] | .05 | AIC: 64182  BIC: 64262 | *t* _NO-SIO_ (62.9) = -.41, *p* =.91  *t* _NO-SSI_ (133.4) = -2.00, *p* =.11  *t* _SIO-SSI_ (114.5) = -1.28, *p* =.41 | 75%  [65.34, 83.12] |

^1^ ID: Subjects; ^2^ Category: Self-Injury Objects, Scenes with Self-Injury;^,3^ *b*-Estimates and standard errors; ^4^ Satterthwaite; ^5^ two-tailed *p*-value; ^6^ 95% bootstrapped CI; ^7^ Adjusted Intraclass Correlation Coefficient; ^8^ Model Fit Indices,^9^ Direct Group Comparisons

**Table 9.** Multilevel Models of Acoustic Startle Reaction, ASR (with significances marked with *)

| **Model** | ***b*-Estimate**^3^ | **Std. Err.** ^3^ | **df**^4^ | ***t*-value** | ***p*-value**^5^ | **CI**^6^ | **ICC**^7^ | **AIC/ BIC**^8^ | **Contrasts**^9^ | **Power**  **[95% CI]** |
| --- | --- | --- | --- | --- | --- | --- | --- | --- | --- | --- |
| **Unconditional Random Intercept Model (Null Model): *ASR ~ 1+(1\|ID*^1^*)*** | | | | | | | | | | |
| ID^1^ | .89 | .01 | 63 | 71.24 | <.001*** | [.87, .92] | .001 | AIC: 5531.5  BIC: 5549.4 | / | / |
| **Conditional Random Intercept Model (M1): *ASR ~ Category + Trial + Startle_Segments + (1\|ID)*** | | | | | | | | | | |
| Intercept  SIO^2^  SSI^2^  Trial  Segments_Startle | 1.30  -.02  -.02  .00  -.02 | .03  .03  .03  .00  .01 | 927.82  2752.11  2748.38  204.65  214.37 | 49.70  -.55  -.89  .08  -3.14 | <.001***  .59  .37  .94  <.001*** | [1.25, 1.35]  [-.08, .05]  [-.07, .03]  [-.01, .01]  [-.03, -.01] | .005 | AIC: 5169.3  BIC: 5210.9 | *t* _NO-SIO_ (2753) = .55, *p* =.85  *t* _NO-SSI_ (2750) = .89, *p* =.65  *t* _SIO-SSI_ (2752) = .11, *p* =.99 | 6%  [2.23, 12.60] |
| **Conditional Random Intercept Model with Interactions (M2): *ASR ~ Category + Trial + Startle_Segments + Trial:Category + Startle:Category + (1\|ID)*** | | | | | | | | | | |
| Intercept  SIO^2^  SSI^2^  Trial  Segments_Startle  Interaction SIO-Trial  Interaction SSI-Trial  Interaction SIO-Segments  Interaction SSI-Segments | 1.31  -.08  -.02  .00  -.02  .00  -.01  .00  .02 | .03  .07  .05  .00  .01  .01  .01  .02  .01 | 1541.51  2806.07  2793.39  630.08  650.80  2790.76  2779.21  2791.11  2777.53 | 40.04  -1.01  -.31  .83  -3.12  -.03  -1.48  .19  1.44 | <.001***  .26  .76  .41  <.001***  .98  .14  .85  .15 | [1.25, 1.37]  [-.21, .06]  [-.12, .08]  [-.00, .01]  [-.04, -.01]  [-.02, .02]  [-.02, .00]  [-.03, .04]  [-.01, .04] | .005 | AIC: 5173.8  BIC: 5239.2 | *t* _NO-SIO_ (2749) = .56, *p* =.84  *t* _NO-SSI_ (2746) = .91, *p* =.63  *t* _SIO-SSI_ (2748) = .12, *p* =.99 | 13%  [7.11, 21.20] |
| **Conditional Random Intercept Random Slope Model (M3): *ASR ~ Category + Trial + Startle_Segments + Trial:Category + Startle:Category + (1+Category\|ID)*** | | | | | | | | | | |
| Intercept  SIO^2^  SSI^2^  Trial  Segments_Startle  Interaction SIO-Trial  Interaction SSI-Trial  Interaction SIO-Segments  Interaction SSI-Segments | 1.31  -.08  -.01  .00  -.03  .00  -.01  .00  .02 | .03  .07  .05  .00  .01  .01  .01  .02  .01 | 2680.50  553.55  786.25  2680.50  2680.50  219.74  353.64  329.06  368.37 | 40.86  -1.21  -.23  .99  -3.37  .00  -1.31  .17  1.26 | <.001***  .23  .82  .32  <.001***  1.00  .19  .87  .21 | [1.25, 1.37]  [-.21, .05]  [-.12, .09]  [-.00, .01]  [-.04, -.01]]  [-.02, .02]  [-.02, .00]  [-.03, .04]  [-.00, .04] | Singularity | AIC: 5176.7  BIC: 5271.7 | *t* _NO-SIO_ (2749) = .53, *p* =.86  *t* _NO-SSI_ (2746) = .79, *p* =.71  *t* _SIO-SSI_ (2748) = .10, *p* =.99 | 19%  [11.84, 28.07] |
| **Conditional Random Intercept Model with Factor Self-Injury (M4): *ASR ~ Category + Trial + Startle_Segments + Post-Self-Injury + Trial:Category + Startle:Category + (1\|ID)*** | | | | | | | | | | |
| Intercept  SIO^2^  SSI^2^  Trial  Segments_Startle  Post-Self-Injury  Interaction SIO-Trial  Interaction SSI-Trial  Interaction SIO-Segments  Interaction SSI-Segments | 1.31  -.08  -.04  .00  -.02  -.05  .00  -.01  .00  .02 | .03  .07  .06  .00  .01  .04  .01  .01  .02  .01 | 1532.60  2804.99  2784.85  631.16  651.76  2782.94  2789.54  2777.94  2789.89  2776.33 | 40.03  -1.12  -.77  .82  -3.12  1.27  -.03  -1.46  .19  1.41 | <.001***  .26  .44  .41  <.001***  .21  .98  .14  .85  .16 | [1.25, 1.37]  [-.21, .06]  [-.15, .07]  [-.00, .01]  [-.04, -.01]  [-.03, .13]  [-.02, .02]  [-.02, .00]  [-.03, .04]  [-.01, .04] | .005 | AIC: 5174.2  BIC: 5245.5 | *t* _NO-SIO_ (2748) = .56, *p* =.84  *t* _NO-SSI_ (2765) = 1.53, *p* =.28  *t* _SIO-SSI_ (2761) = .79, *p* =.71 | 22%  [14.22, 31.39] |

^1^ ID: Subjects; ^2^ Category: Self-Injury Objects, Scenes with Self-Injury;^,3^ *b*-Estimates and standard errors; ^4^ Satterthwaite; ^5^ two-tailed *p*-value; ^6^ 95% bootstrapped CI; ^7^ Adjusted Intraclass Correlation Coefficient; ^8^ Model Fit Indices,^9^ Direct Group Comparisons

**Table 10.** Multilevel Models of the Skin Conductance Response, SCR (with significances marked with *)

| **Model** | ***b*-Estimate**^3^ | **Std. Err.**^3^ | **df**^6^ | ***t*-value** | ***p*-value**^4^ | **CI**^6^ | **ICC**^7^ | **AIC/ BIC**^8^ | **Contrasts (two-tailed)** ^9^ | **Power**  **[95% CI]** |
| --- | --- | --- | --- | --- | --- | --- | --- | --- | --- | --- |
| **Unconditional Random Intercept Model (Null Model): *SCR ~ 1+(1\|ID*^1^*)*** | | | | | | | | | | |
| ID^1^ | .21 | .02 | 58.62 | 8.32 | <.001*** | [.16, .26] | .17 | AIC: 6622.9  BIC: 6642.8 | / | / |
| **Conditional Random Intercept Model (M1): *SCR ~ Category + Trial + Startle + (1\|ID)*** | | | | | | | | | | |
| Intercept  SIO^2^  SSI^2^  Trial  Startle | .21  .04  .010  .00  .14 | .03  .02  .01  .00  .01 | 91.45  5562.63  5562.59  5563.09  5562.53 | 7.51  2.50  7.80  -11.56  12.69 | <.001***  .01*  <.001***  <.001***  <.001*** | [.15, .26]  [.01, .07]  [.07, .12]  [-.00, -.00]  [.12, .16] | .18 | AIC: 6286.6  BIC: 6333.1 | *t* _NO-SIO_ (5567) = -2.50, *p* =.033*  *t* _NO-SSI_ (5567) = -7.80, *p* < .001***  *t* _SIO-SSI_ (5567) = -3.13, *p* = .005** | 100%  [96.38, 100] |
| **Conditional Random Intercept Model with Interactions (M2): *SCR ~ Category + Trial + Startle + Trial:Category + Startle:Category + (1\|ID)*** | | | | | | | | | | |
| Intercept  SIO^2^  SSI^2^  Trial  Startle  Interaction SIO-Trial  Interaction SSI-Trial  Interaction SIO-Startle  Interaction SSI-Startle | .18  .04  .17  .00  .14  .00  .00  -.01  .01 | .03  .04  .03  .00  .02  .00  .00  .03  .02 | 121.13  5564.47  5561.67  5561.50  5559.09  5568.13  5563.53  5559.64  5558.98 | 6.11  1.02  6.36  -6.16  8.81  .17  -4.00  -.19  -.51 | <.001***  .31  <.001***  <.001***  <.001***  .86  <.001***  .85  .61 | [.12, .24]  [-.04, .11]  [.12, .23]  [-.00, .00]  [.11, .17]]  [-.00, -.00]  [-.00, .00]  [-.07, .06]  [-.04, -.06] | .18 | AIC: 6276.2  BIC: 6349.2 | *t* _NO-SIO_ (5563) = -2.46, *p* =.037*  *t* _NO-SSI_ (5563) = -7.81, *p* < .001***  *t* _SIO-SSI_ (5563) = -3.18, *p* = .004** | 100%  [96.38, 100] |
| **Conditional Random Intercept Random Slope Model (M3): *SCR ~ Category + Trial + Startle + Trial:Category + Startle:Category + (1+Category\|ID)*** | | | | | | | | | | |
| Intercept  SIO^2^  SSI^2^  Trial  Startle  Interaction SIO-Trial  Interaction SSI-Trial  Interaction SIO-Startle  Interaction SSI-Startle | .18  .04  .18  .00  .14  .00  .00  -.01  .01 | .03  .04  .03  .00  .02  .00  .00  .03  .02 | 115.29  1627.97  362.88  5516.97  5496.44  5428.35  5514.71  5494.75  5494.45 | 6.56  1.02  5.58  -6.29  8.94  .19  -4.08  -.18  .50 | <.001***  .31  <.001***  <.001***  <.001***  .85  <.001***  .86  .62 | [.13, .24]  [-.04, .11]  [.12, .24]  [-.00, -.00]  [.11, .17]]  [-.00, .00]  [-.00, -.00]  [-.07, .06]  [-.04, -.06] | .19 | AIC: 6228.7  BIC: 6334.9 | *t* _NO-SIO_ (62.7) = -2.25, *p* =.07  *t* _NO-SSI_ (62.8) = -4.73, *p* < .001***  *t* _SIO-SSI_ (62.6) = -2.91, *p* = .014* | 98%  [92.96, 99.76] |
| **Conditional Random Intercept Model with Factor Self-Injury (M4): *SCR ~ Category + Trial + Startle + Post-Self-Injury + Trial:Category + Startle:Category + (1+Category\|ID)*** | | | | | | | | | | |
| Intercept  SIO^2^  SSI^2^  Trial  Startle  Post-Self-Injury  Interaction SIO-Trial  Interaction SSI-Trial  Interaction SIO-Startle  Interaction SSI-Startle | .18  .04  .17  .00  .14  .01  .00  .00  -.01  .01 | .03  .04  .03  .00  .02  .02  .00  .00  .03  .02 | 117.47  2793.93  601.19  5510.26  5493.13  5491.10  5412.98  5519.34  5496.25  5491.81 | 6.61  1.04  5.33  -6.25  8.92  .45  .20  -4.11  -.18  -.49 | <.001***  .30  <.001***  <.001***  <.001***  .65  .84  <.001***  .86  .62 | [.13, .24]  [-.04, .11]  [.11, .24]  [-.00, -.00]  [.11, .17]]  [-.03, .05]  [-.00, .00]  [-.00, -.00]  [-.07, .06]  [-.04, -.06] | Singularity | AIC: 6230.5  BIC: 6343.3 | *t* _NO-SIO_ (62.6) = -2.47, *p* =.04*  *t* _NO-SSI_ (113) = -4.23, *p* < .001***  *t* _SIO-SSI_ (98.3) = -2.03, *p* = .11 | 100%  [96.38, 100] |

^1^ ID: Subjects; ^2^ Category: Self-Injury Objects, Scenes with Self-Injury;^,3^ *b*-Estimates and standard errors; ^4^ Satterthwaite; ^5^ two-tailed *p*-value; ^6^ 95% bootstrapped CI; ^7^ Adjusted Intraclass Correlation Coefficient; ^8^ Model Fit Indices,^9^ Direct Group Comparison
